# Mislabeled and Misunderstood: Large Mammal Distribution Underscores Ecological Significance of Agro‐Pastoral “Wastelands” in India's Deccan Peninsula

**DOI:** 10.1002/ece3.72937

**Published:** 2026-01-12

**Authors:** Iravatee Majgaonkar, Anish Paul, Sushma Sharma, Indrajeet Ghorpade

**Affiliations:** ^1^ Manipal Academy of Higher Education Manipal India; ^2^ Ashoka Trust for Research in Ecology and the Environment Bengaluru India; ^3^ Wildlife Conservation Society‐India Bengaluru India; ^4^ Centre for Wildlife Studies Bengaluru India; ^5^ Wildlife Biology and Conservation Program, National Centre for Biological Sciences Bengaluru India; ^6^ Biodiversity and Ecosystems Ecology Research Laboratory, National Centre for Biological Sciences Bengaluru India; ^7^ Department of Ecology and Evolutionary Biology University of Michigan Ann Arbor USA; ^8^ Independent Researcher Nagarabhavi Bengaluru India; ^9^ Deccan Conservation Foundation Koppal Karnataka India

**Keywords:** carnivores, Deccan peninsula, human‐use landscapes, occupancy, open natural ecosystems, wastelands

## Abstract

Multi‐use landscapes are now recognized for their value in supporting biodiversity and aiding species conservation, including charismatic megafauna. However, semi‐arid open‐canopy human‐use landscapes have faced multiple anthropogenic stressors over the past centuries and have received meager conservation attention, especially in South Asia. A growing body of evidence suggests that such ecosystems, even with intermittent human use, can provide habitats for globally threatened species and support their conservation. To understand the role of semi‐arid multi‐use landscapes in supporting populations of large‐bodied wildlife in India's Deccan Peninsula, we used key informant interviews with pastoralists and a single‐season single‐species occupancy modeling framework and examined the distribution of three species: striped hyena *Hyaena hyaena* , sloth bear *Melursus ursinus* , and blackbuck *Antilope cervicapra* . Hyena, sloth bear and blackbuck occupied 52%, 26% and 63% of the landscape, respectively, despite the absence of intensively managed protected areas. Conservative estimates suggest that Indian gray wolf ( *Canis lupus pallipes* ) was present in at least 76% of the landscape. ONEs favored occupancy of hyena and sloth bear, while low‐intensity agriculture supported blackbuck presence. Our results highlight the conservation potential of agro‐pastoral landscapes and challenge the narrative of characterizing semi‐arid open ecosystems as “wastelands”. We also demonstrate how experiential knowledge of communities can be applied to ecological research when using traditional methods is infeasible. Under global change scenarios, misclassification and mismanagement of critical socio‐ecological systems, such as the ONEs of Deccan Peninsula, will not only jeopardize the survival of populations of threatened species but also weaken the land‐sharing potential of these regions.

## Graphical abstract

A large part of India's Deccan peninsula consists of agricultural lands interspersed with open ecosystems that support megafauna populations. We examined the distribution of four large mammals: blackbuck, striped hyena, sloth bear and the Indian wolf in agro‐pastoral landscapes of north Karnataka's Koppal district using an interview‐based occupancy framework. While blackbuck presence was supported by low‐intensity agriculture and hyena and sloth bear presence was driven by presence of open ecosystems, conservative estimates for Indian wolves showed that they were present in at least 76% of the district's area. We argue that open ecosystems‐agriculture matrices in the Deccan be recognized for their ability to support charismatic megafauna and that they should not be mislabeled as “wastelands” in developmental planning.

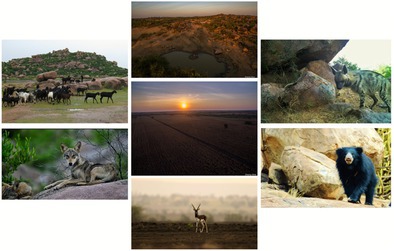

## 1 Introduction

Large carnivores and herbivores, despite their ecological significance and cultural veneration, have experienced drastic declines in their global distribution over the past century of the Anthropocene (Ripple et al. 2015, 2014). Until recently, conservation of these species has primarily revolved around earmarking patches of “intact” natural habitats, thought to be of high ecological value, as inviolate protected areas (Carter and Linnell 2016; Krishnadas et al. 2018; Woodroffe 2000). However, protected areas account for only 16% of the global landmass, and in developing nations, the placement of protected areas is often unjust towards communities (Barr et al. 2011; Ghosh‐Harihar et al. 2019; Palfrey et al. 2022; Srivathsa et al. 2023). Moreover, even the placement of protected areas does not adequately cover all ecosystems and is often opportunistic and biased towards areas with low human population density (Baldi et al. 2017; Joppa and Pfaff 2009; Sayre et al. 2020). Although protected areas may play crucial roles in supporting populations of endangered flora and fauna and preserving essential species interactions, they fall short of conserving species that are wide‐ranging and adaptable to several small and large‐scale anthropogenic activities (Brashares et al. 2001; Ghosh‐Harihar et al. 2019; Packer et al. 2013; Warrier et al. 2020). But conservation research and practice now have increasingly encompassed areas beyond contiguous native habitats and focused on the co‐occurrence of large wildlife with humans in multi‐use landscapes for understanding the ecology and distribution of adaptable species (Athreya et al. 2013; Bartoń et al. 2019; Bateman and Fleming 2012; Madhusudan et al. 2015; Paul et al. 2024; Suraci et al. 2020; Valeix et al. 2012).

Arid and semi‐arid human‐use landscapes in the global south are no exception when it comes to supporting populations of threatened species (Athreya et al. 2013; Brown et al. 2023; Connolly et al. 2021; Farhadinia et al. 2018; Kannan et al. 2022; Khan et al. 2025; Majgaonkar et al. 2019; Srivathsa et al. 2020). While some semi‐arid human‐use landscapes with low human densities can serve as connectivity corridors between source populations in protected areas (Kabir et al. 2017; Rezaei et al. 2022), some have documented their gross effectiveness in the conservation of threatened species (Mohammadi, Almasieh, et al. 2021; Mohammadi, Lunnon, et al. 2021; Ogutu et al. 2017). However, not all semi‐arid regions in the global south have similar socio‐ecological conditions and this is expected to shape species ecology and conservation in these regions. For instance, regions in West Asian and sub‐Saharan African countries have a considerable area with low human density, which is either unsuitable for cropland development or not yet intensively cultivated (Baldi et al. 2017; Cunningham and Beazley 2018; Sayre et al. 2020; Watson et al. 2016) and may allow for effective land‐sharing for biodiversity conservation (Karimi et al. 2023; Kiffner et al. 2020).

**FIGURE 1:**
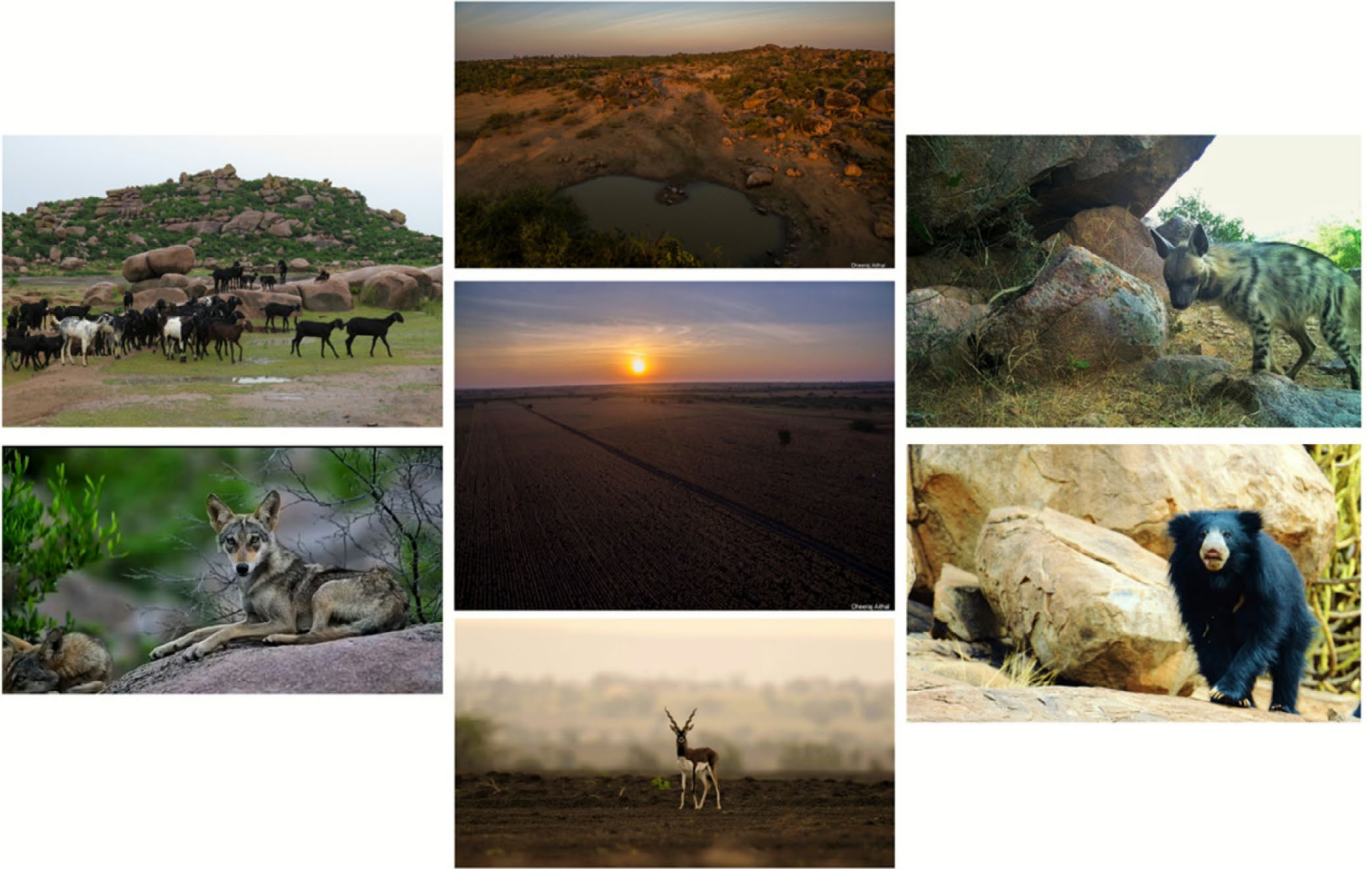
Semi‐arid agro‐pastoral open ecosystems of Koppal, typical of the Deccan Peninsula, supports a wide range of native large mammals, livestock and human livelihoods. Photo credits: Indrajeet Ghorpade & Dheeraj Aithal.

In semi‐arid regions of India however, agricultural land tenures dominate and human densities are much higher. Populations of species like the blackbuck ( *Antilope cervicapra* ), chinkara ( *Gazella bennettii* ), Indian wolf ( *Canis lupus pallipes* ), sloth bear ( *Melursus ursinus* ), and striped hyena ( *Hyaena hyaena* ), which depend on native semi‐arid habitats like “Open Natural Ecosystems” (ONEs; Madhusudan and Vanak 2023) persist to differing extents in these mixed‐use semi‐arid regions (Jangid et al. 2023; Majgaonkar et al. 2019; Manoj Kumar et al. 2025; Rahmani 1990). Assessments of the potential of such landscapes to support animal populations are more frequently undertaken across regions that contain protected area networks, with areas managed at differing intensities (Gubbi et al. 2020; Manoj Kumar et al. 2025; Puri et al. 2022). Therefore, biophysical drivers of large mammal assemblages in regions devoid of intensively managed protected areas and dominated by agriculture‐ONE matrices are poorly known. Additionally in India, non‐forested arid and semi‐arid regions (Figure 1) are governed primarily from the perspective of either agricultural production or forestry (Baka 2017; Department of Land Resources, National Remote Sensing Centre 2019; Hanumantha Rao 1994; Watve et al. 2021). However, these landscapes have historically neither supported year‐round agrarian activities nor a dense forest cover (Ratnam et al. 2016, 2011; Riedel et al. 2021). They are often misclassified as “wastelands” (Sankaran and Ratnam 2013; Whitehead 2010) or perceived as “degraded forests” (Lahiri et al. 2023; Ratnam et al. 2016, 2011) and this has either constrained conservation interventions in these landscapes or threatened the ecological integrity of these regions, both of which are essential for supporting wide‐ranging species. This has undeniably led to rapid land use and tenurial changes (Tian et al. 2014; Whitehead 2010), which have been structural or functional in nature. These changes have reduced the contiguity of ONEs to an extent where 94% of ONE patches in India are now between 1 and 100 ha in size (Madhusudan and Vanak 2023). Interestingly, in some regions, the same changes have contributed to the replacement of species adapted to semi‐arid landscapes with newer species assemblages, particularly those that adapt to irrigated agricultural landscapes (Athreya et al. 2013; Majgaonkar et al. 2019; Rahmani and Soni 1997). It thus becomes essential to undertake research and monitoring of species populations in these rapidly transforming landscapes to understand how ecological change may impact land sharing between humans and wildlife.

In India, the agro‐pastoral non‐forested landscapes of the Deccan peninsula have remained a geographic gap in biodiversity assessments of semi‐arid regions. This is likely because of the dominance of private agricultural tenures over contiguous native habitats, the latter especially under the forest department (Ghosh‐Harihar et al. 2019), high soil erosion rates (Singh et al. 1992) leading to depauperation and relatively low diversity of wide‐ranging species (Srivathsa et al. 2022; Sudhakar Reddy et al. 2016). Additionally, the neglect also indicates a form of presentism, wherein current land use characteristics have obscured the historically richer ecological characteristic of these landscapes. Thus, we lack baseline knowledge about how fragmented semi‐arid agro‐pastoral ONEs support large mammal assemblages in these regions, while being situated in a matrix of human‐use landscapes. This becomes highly pertinent as these landscapes are being transformed to achieve developmental and climate change mitigation goals through intensification of agriculture, afforestation and green‐energy initiatives (Baka 2017; Whitehead 2010). Koppal district in the state of Karnataka is deemed suitable for such enquiry as it covers large swathes of agriculture‐ONE matrix, a characteristic of the Deccan peninsula where land use is dominated by private agricultural tenure. Our objective was to understand the relative role of ONEs (most of which are classified as wastelands under land use planning; Department of Land Resources, National Remote Sensing Centre 2019) and agricultural lands in sustaining large mammals which are wide‐ranging and whose persistence hinges on land sharing in semi‐arid areas.

In human‐dominated multi‐use areas, such as the agro‐pastoral landscapes of the Deccan Peninsula, detecting animal presence using traditional methods such as sign surveys and camera traps becomes difficult due to heavy anthropogenic traffic, erasure of animal signs and the risk of theft (Paul et al. 2024). Additionally, using camera traps in such landscapes raises ethical concerns about photographing people without their consent, leaving them vulnerable to digital surveillance (Simlai and Sandbrook 2025). Interview‐based detection methods, paired with well‐suited modeling frameworks, are hence useful to understand animal distributions in such shared landscapes (Kachel et al. 2022; Majgaonkar et al. 2019; Miller et al. 2013; Pillay et al. 2014; Ratnayeke, van Manen, Pieris, and Pragash 2007). Extensive pastoralism is one of the major livelihoods in our study site and is largely dependent on ONEs. Movement of these nomadic pastoralists through the landscape enable them to encounter and observe wildlife very often (Madsen et al. 2020). Hence, we leveraged pastoralists' experiential knowledge of animal occurrence and employed key informant surveys with them to investigate the distribution and space‐use patterns of five species whose populations are threatened in India—Indian leopard ( *Panthera pardus fusca* ; IUCN status: Near Threatened), Indian gray wolf (IUCN status: Vulnerable), striped hyena (IUCN status: Near Threatened), sloth bear (IUCN status: Vulnerable) and blackbuck (IUCN status: Least Concern).

## 2 Materials & Methods

### 2.1 Study Site

**FIGURE 2:**
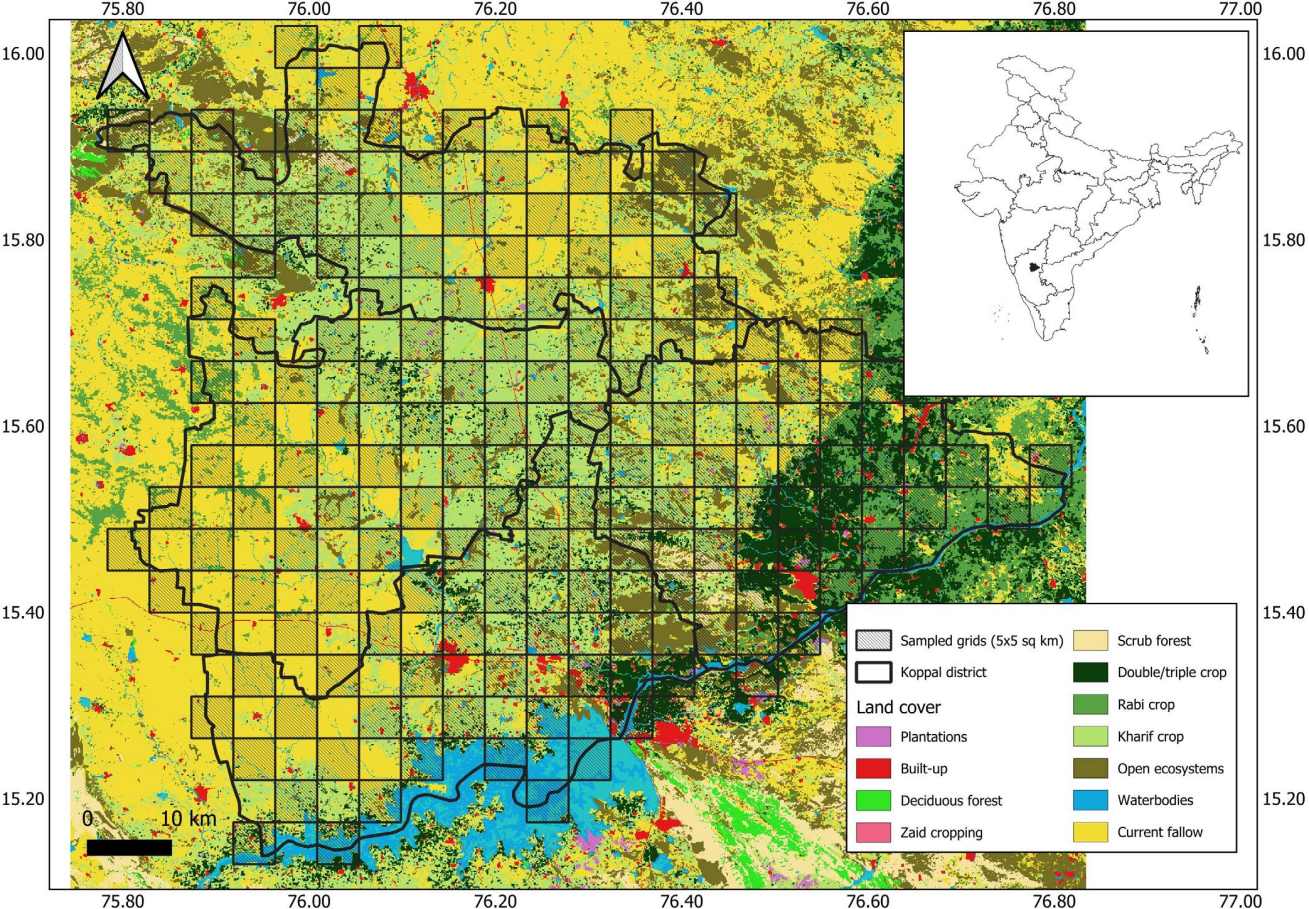
Map showing land cover in Koppal district overlaid with the grid design for our study. The land cover map of Koppal has been sourced from Madhusudan and Vanak (2023).

We studied the distribution of carnivores in Koppal district in the state of Karnataka, which lies between 15.46797° N 75.78361° E, 15.53451° N 76.81866° E and 16.02940° N 76.03027° E, 15.12940° N 76.006105° E, and is part of the Deccan Peninsula (Figure 2). The Deccan is a distinct semi‐arid biogeographic zone covering the south‐central parts of the Indian peninsula, supporting native habitats such as deciduous forests, open savanna grasslands, and rocky scrublands (Banerjee et al. 2022). The annual rainfall received ranges from 500 to 700 mm, and census data records a 5.73% forest cover in the district, much of which is scrubland (Directorate of Census Operations 2011). Koppal district has an area of 5570 km^2^, out of which primary ONEs cover 8%, that is, 448 km^2^ (Madhusudan and Vanak 2023), and are characterized by rocky inselbergs, open savannas and scrub vegetation (Koulgi and Madhusudan 2024) (Figure 1). Koppal has two distinct soil types: black clayey soils and red sandy soils. These support paddy, jowar and bajra (millets), which are the principal food crops of the district, in addition to commercial crops such as oilseeds and cotton. Small (sheep and goat) and large (cow) ruminant pastoralism is a primary livelihood in the district, which is seasonally dependent on the ONEs and agricultural lands. The district population is 13.89 lakh (Directorate of Census Operations 2011). As of 2021, 74.63% of Koppal's area is sown for at least one cropping cycle and de jure “permanent pasture” covers 3.23% of the district (Directorate of Economics and Statistics 2021).

### 2.2 Field Sampling Method

We divided the entire Koppal district into 5 km × 5 km grid cells to conduct key informant interviews with pastoralists. The grid cell was bigger than the average home range sizes of Indian leopards (Odden et al. 2014), striped hyenas (Athreya et al. 2013), sloth bears (Joshi 1995; Ratnayeke, van Manen, and Padmalal 2007), and blackbucks (Mahato et al. 2010), but was much smaller compared to that of Indian wolves (Habib 2007; Jhala et al. 2022; Jhala and Giles 1991; Sadhukhan et al. 2024). Thus, the cell size was adequate to examine site occupancy for leopards, hyenas, sloth bears, and blackbucks, and habitat use for wolves.

Pastoralist systems in this landscape involve people taking their animals on daily grazing routes in different directions around the village or camp to access fodder and water. This allows them to regularly cover native habitats around their villages, increasing their likelihood of encountering wild animals (Madsen et al. 2020). To keep the survey effort manageable, we sampled every alternate grid (Figure 2) to obtain detection data for all five focal species between December 2017 and March 2018. If more than half of a grid cell was situated outside the district or was covered with waterbodies, it was not sampled. Using georeferenced Survey of India, 2010 toposheets (scale: 1:50,000), all village names were digitized in QGIS 2.16.0. The ArcView 3.2 software was then used to generate physical maps for each sampling grid and its eight neighboring grids, including all village names and locations on the map, so that the survey team could refer to these maps while conducting the interview. Each sampling grid (focal grid) was divided into four sub‐grids to accurately describe the boundaries to key informants. In case focal grids did not contain villages/settlements, the surveyors interviewed pastoralists in neighboring grids, and responses were elicited for the focal grid. The survey team visited villages in each grid to locate key informants, either by enquiring inside the villages or by approaching pastoralist camps in crop fields around the village. Interviews were conducted in two grids daily, between 0630 and 1100 h.

Responses were primarily collected from a single respondent at a time; however, in some cases, two pastoralists were interviewed together, and their responses were combined into a single entry. If the interviewee pastoralist was in a group containing non‐pastoralists, others were requested not to respond during the interview. Because pastoralists move camps and graze livestock in a certain radius around it, we confirmed the geographical extent of the key informant's movement over the last year in and around the focal grid to assess the proportion of the focal grid they covered regularly. We did not conduct an interview if the pastoralist had not covered at least two sub‐grids within the focal grid. Depending on the number of villages inside a focal grid, we ended up sampling a variable number of villages per grid to achieve a minimum of four interviews per grid. If a pastoralist covered at least three of the four subgrids inside a focal grid, the interview was considered an independent replicate. In cases where they covered two of the four subgrids, it was ensured that another pastoralist, who covered the other one or two subgrids, was interviewed. These two interviews were then combined and treated as a single response. We obtained approval from the Human Subjects Committee of Centre for Wildlife Studies and Wildlife Conservation Society, India, following which we conducted all surveys in the local language, *Kannada*, after obtaining verbal consent from respondents.

### 2.3 Occurrence Data Collation

We initially decided to employ false‐positive occupancy models to estimate detection probability (*p*) and occupancy (Ψ), as interview data is likely to have some possibility of false‐positive detection of species (Madsen et al. 2020; Pillay et al. 2014; Royle and Link 2006). Thus, we aimed to classify all the reported detections into “CERTAIN” (zero probability of false‐positive detections to occur) and “UNCERTAIN” (non‐zero probability of false‐positive detections to occur) categories, following the detection framework of type III false‐positive occupancy models. Each survey began by asking pastoralists to list all wildlife species they had seen in the focal grid. We then showed photos of our five focal species: Indian leopard, Indian gray wolf, striped hyena, sloth bear and blackbuck. The leopard photo was paired with a photo of a tiger and the wolf photo with a jackal, species of similar appearances as our focal species to further investigate the credibility of each detection for the false‐positive model. A detection was considered “CERTAIN” only if the respondent could correctly identify all species of the paired sets as well as the focal species, without requiring any hint from the interviewer (Figure S1). If the respondent was successful in identifying the species of the paired sets but needed hints from the interviewer about the morphology and behavior of the focal species to identify it, the detection was recorded as “UNCERTAIN” (Figure S1). If the respondent failed to identify one or more species of the paired set but could identify the focal species with or without any hint, the detection was also considered as “UNCERTAIN” (Figure S1). However, despite getting a hint from the interviewer, if the respondent failed to identify one or more of the focal species, it was recorded as “Failed to ID” and the interview was not considered as a replicate while modeling occupancy for that species (Figure S1). We enquired if the pastoralist had spotted the species (dead or alive) in the focal grid in the previous year alone. We recorded the village name, cardinal position of the detection site from a closest village, approximate distances from nearby villages and prominent landscape features to confidently ascribe the detection to the focal grid. We also noted down descriptions about the sighting, such as number of individuals, their behavior, time of day, pastoralist activity at the time and surrounding habitat features. In cases where two pastoralists were interviewed together, we ensured that the above‐mentioned identification criteria were fulfilled for each pastoralist for all the focal species. Following this protocol, we constructed detection histories for all five species for each sampled grid, where “0,” “1” and “2” referred to non‐detection, uncertain detection, and certain detection, respectively (irrespective of the number of sightings and individuals spotted) (Majgaonkar et al. 2025). However, despite taking utmost care and due diligence to ensure that recorded detections pertained to the focal cell from the past 1 year, there remains a minor non‐zero probability of error in spatiotemporal assignment of these detections. This could be due to factors like deliberate falsification or accidental misestimation of distance by respondents, and/or inadvertent mistakes by the surveyor in calculation of distances. However, we also considered a reported animal sighting as a non‐detection for the focal cell when sightings fell either on the border or immediately outside it. Thus, we have strong reason to assume that our method was effective and conservative in assigning detections to focal cells vs. neighboring cells.

### 2.4 Covariate Extraction

For each interview (with either an individual or a pair of pastoralists), we recorded (i) “years of experience with herding” and (ii) proportion of the grid that the pastoralist covered (Brittain et al. 2020; Petracca et al. 2018). We used these two covariates to model detection probability. The number of interviews where two shepherds were together was negligible (4/235 for hyena, 10/488 for sloth bear, 6/491 for blackbuck, 9/424 for leopard, 10/456 for wolf). Such few samples were unlikely to influence the detection process, and hence we did not include “number of respondents” as a covariate for detection. A few indirect species‐specific habitat covariates, likely to influence abundance (and hence, detection), were also included as detection covariates (Table 1). In cases where two responses were combined into one interview, we retained the experience of the shepherd with longer herding experience. Grid area covered by single or combined pastoralists was converted into one of two values: “0” (when three out of the four subgrids were covered) and “1” (when all the subgrids were covered) (Majgaonkar et al. 2025). In cases where the pastoralists approximated their years of experience, we took an average of the reported range of years (Majgaonkar et al. 2025).

**TABLE 1:** List of covariates used to model detection probability (*p*) and occupancy probability (Ψ) of five species.

Covariate	Covariate type	Expected relationship				
		Leopard^a^	Wolf^b^	Hyena^c^	Sloth bear^d^	Blackbuck^e^
Large and small‐bodied livestock biomass	Ψ	+	NA	+	NA	NA
Small‐bodied livestock biomass	Ψ	NA	+	NA	NA	NA
Road length	Ψ	−	−	−	−	−
Area under double/triple crop	Ψ & *p*	+	NA	+	NA	NA
Area under kharif crop	Ψ & *p*	NA	+	NA	+	+
Area under ONE	Ψ & *p*	+	+	+	+	+
Grid‐covered by the respondent	*p*	+	+	+	+	+
Years of shepherding experience of respondent	*p*	+	+	+	+	+

*Note:* “+” represents an expected positive relationship, “−” represents an expected negative relationship.

^a^ Athreya et al. (2015); Athreya et al. (2013); Gubbi et al. (2020); Naha et al. (2021).

^b^ Habib (2007); Habib et al. (2021); Jhala et al. (2022); Mukherjee et al. (2021); Singh and Kumara (2006).

^c^ Athreya et al. (2013); Mukherjee et al. (2021); Singh et al. (2010); Singh et al. (2014).

^d^ Bargali et al. (2004); Puri et al. (2015); Ratnayeke, van Manen, Pieris, and Pragash (2007); Rot et al. (2023).

^e^ Asif and Modse (2016); Delu et al. (2024); Jhala and Isvaran (2016); Krishna et al. (2016).

To model occupancy probability, we extracted three site‐level covariates for all the sampled and unsampled grids—(i) area under three different land covers (in km^2^), viz. kharif cropping (monsoon cropping), double/triple cropping (irrigated cropping), and ONEs (ii) major road network length (in km) (iii) total biomass of small and large‐bodied domestic livestock (in kg) (Majgaonkar et al. 2025). For land cover classes, we used 2015–2016 1:50,000 LULC raster from Indian Space Research Organization's Bhuvan portal (https://bhuvan‐app1.nrsc.gov.in/thematic/thematic/index.php). To extract area under ONEs for each grid, we used the raster developed by Madhusudan and Vanak (2023) and counted all pixels with probability values > 0.5 as ONEs. Road lengths from the year 2019 were obtained from the open‐access datasets by Geofabrik GmbH and OpenStreetMap contributors (https://download.geofabrik.de/asia/india/southern‐zone.html). Village‐wise numbers of the four common livestock types—cow, buffalo, sheep, and goat (Department of Animal Husbandry and Veterinary Services 2019)—were multiplied by their average weights available in the literature to estimate biomass (Hussain et al. 2019; Mundinamani et al. 2024; Siddalingamurthy et al. 2017). Livestock‐wise biomass was added to obtain village‐wise total livestock biomass. In cases where a village was spread across multiple grids, we divided the livestock numbers from that village among the respective grids in proportion to the village's area in each grid. The proportions were then added to obtain grid‐level livestock biomass, which was calculated separately for small‐bodied livestock (sheep and goats) and large‐bodied livestock (cows and buffaloes). All covariates were extracted using QGIS 3.22.9 and R version 4.2.2 (R Core Team 2024). We expected that ONEs in the form of rocky outcrops and savanna grasslands would support occupancy of all species because of their ruggedness and the infrequent presence of humans. Similarly, we expected monsoon cropping to support open‐habitat species like wolves and blackbucks and sloth bears because of low human presence for much of the year. Irrigated cropping, because of its densely vegetated cover, was expected to support leopard and hyena presence while road length was thought to have a negative impact on the occupancy of all species because of increased human movement. We expected livestock biomass to support leopard, wolf, and hyena occupancy in the study area because it is a major part of their diet outside protected areas. Specific predictions for each species are provided in Table 1.

### 2.5 Occupancy Analysis

Our false‐positive models exhibited convergence errors and yielded very low false‐positive estimates, associated with high standard errors. Hence, we shifted to using standard single‐season single‐species occupancy models to model occupancy for all five species in the landscape (MacKenzie et al. 2018, 2002). As single‐season single‐species occupancy models cannot correct for false‐positive detections but can consider the false‐negatives, we converted the detection history of all the species in a way so that we could minimize the chances of having false‐positive detections in the data. All non‐detections (0) of the false‐positive detection history were retained as non‐detections (0). The uncertain detections (1) of the false‐positive detection history were also converted to non‐detections (0) to avoid false‐positive errors. And finally, all certain detections (2) of the false‐positive detection history were converted to detections (1) to be modeled in a single‐season single‐species occupancy model framework (Majgaonkar et al. 2025).

All covariates were standardized using *z*‐scores and examined for cross‐correlations; covariates with a Pearson's correlation (*r*) of ≥ |0.6| were excluded from the same model. We used a two‐step process to predict the parameters of interest: *p* and Ψ (Doherty et al. 2012). We first performed model selection for *p* while keeping the null model of Ψ constant. We fitted a global additive model of *p* and simplified it by excluding the covariates whose SE values were greater than or equal to their corresponding β estimates. We then refitted the simplified global model and built competing models with singular and additive effects of the remaining covariates. A goodness‐of‐fit test was conducted on the simplified global model to test for overdispersion (ĉ > 1). The model with the best relative fit for *p* was chosen using Quasi Akaike Information Criterion corrected for small sample size (QAICc), incorporating the value of the overdispersion parameter (ĉ) from the simplified global model (Burnham and Anderson 2002). The best‐fit model was kept constant while building models to estimate Ψ. In the case of Ψ, we used a similar model‐building approach where we simplified the additive global model by comparing β estimates of each parameter with their corresponding SE values and subsequently built singular and all possible additive combinations of the remaining covariates. If any model built using this approach exhibited any convergence issues, it was not considered for model comparison (one model for hyena detection and another for hyena occupancy). The goodness‐of‐fit tests and model comparisons were performed following the similar approach as that of detection probability (*p*). The top models were selected based on a ΔQAICc ≤ 2.0 from the best‐performing model. We predicted occupancy estimates for the whole district by model averaging across the models with ΔQAICc ≤ 2. We excluded predicted occupancy estimates for five grid cells as more than 3/4th of these cells were covered by waterbodies. All analyses were done using the package “*unmarked*” in R version 4.4.2 (Fiske and Chandler 2011; R Core Team 2024).

## 3 Results

**FIGURE 3:**
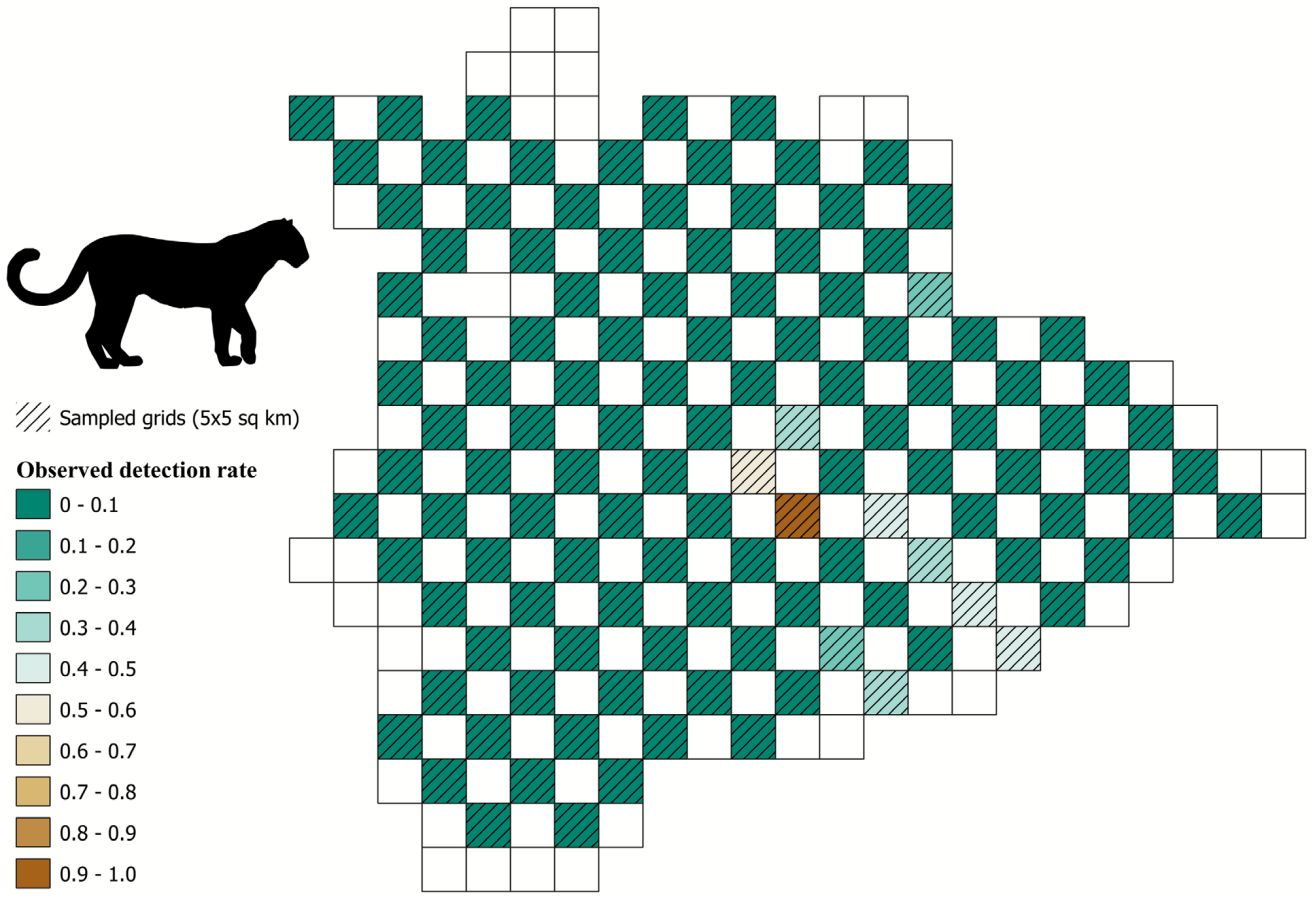
Map depicting the spatial distribution of the observed detection rate (number of detections/total number of replicates) of leopard in the study area.

**FIGURE 4:**
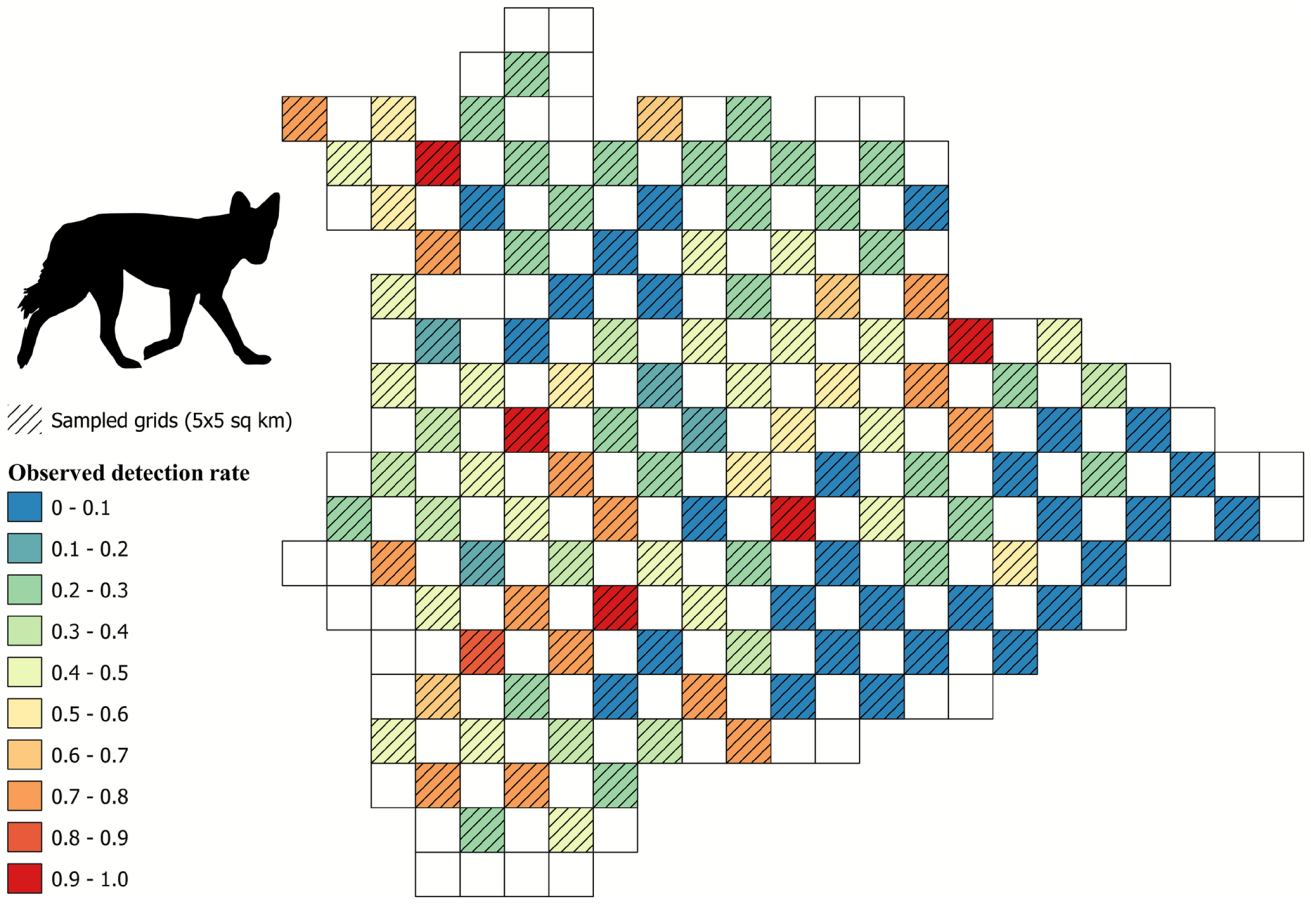
Map depicting the spatial distribution of the observed detection rate (number of detections/total number of replicates) of wolves in the study area.

We created detection/non‐detection matrices for the five focal species, with data from 118 sites for leopard (424 interviews, 1–6 interviews per site), 119 sites for wolf (456 interviews, 1–6 interviews per site), 101 sites for hyena (235 interviews, 1–5 interviews per site), 119 sites for sloth bear (488 interviews, 1–6 interviews per site), and 119 sites for blackbuck (491 interviews, 1–7 interviews per site). Leopard presence was detected in 10 sites (naive occupancy 0.09, Figure 3), wolf presence was detected in 90 sites (naive occupancy 0.76, Figure 4), hyena presence was detected in 21 sites (naive occupancy 0.21), sloth bear presence was detected in 14 sites (0.12), and blackbuck presence was detected in 45 sites (naive occupancy 0.39). We did not use occupancy models with a covariate structure to predict the spatial distribution of leopard and wolf, as the null occupancy was very low (Table 2, Figure 3) and very high (Table 2, Figure 4), respectively, for these two species. We compared three models for hyenas (Table 3), four models for sloth bears (Table 4), and four models for blackbucks (Table 5) to predict their occupancy probability in Koppal. No single model supported the data substantially better than the other competing models. Two models for hyena (cumulative QAIC wt. = 0.93, Table 3), four models for sloth bear (cumulative QAIC wt. = 1.00, Table 4), and two models for blackbuck (cumulative QAIC wt. = 0.91, Table 5) ranked within ΔQAIC ≤ 2, thus indicating that these models were similar in their performance to predict occupancy of the corresponding species.

**TABLE 2:** *ꞵ* estimates and associated standard error values for the single‐season single‐species Null model (Ψ(.), *p*(.)) and all covariates from the top models of the candidate set of models in which they first appear.

Covariate	Leopard	Wolf	Hyena	Sloth Bear	Blackbuck
Null	0.11 (0.04)	0.94 (0.05)	0.70 (0.25)	0.18 (0.06)	0.44 (0.5)
Large and small‐bodied livestock biomass	NA	NA	—	NA	NA
Small‐bodied livestock biomass	NA	NA	NA	NA	NA
Major road length	NA	NA	1.05 (0.72)	0.85 (0.41)	−1.20 (0.40)
Area under double/triple crop (irrigated agriculture)	NA	NA	—	NA	NA
Area under kharif crop (monsoonal agriculture)	NA	NA	NA	—	1.30 (0.99)
Area under ONE	NA	NA	1.66 (1.58)	1.06 (0.87)	—

*Note:* “—” represents that the covariate was not used in modeling the occupancy of the focal species, as the SE was greater than the *ꞵ* estimate for the corresponding covariate in the global occupancy model of the species.

**TABLE 3:** Additive models run to predict detection probability (*p*) and occupancy probability (Ψ) for hyena. Detection probability (*p*) was modeled by keeping the Null structure for occupancy probability (Ψ) constant.

	*K* (#Parameters)	QAICc	ΔQAICc	QAICc weight	Cumulative weight	Quasi‐log likelihood
Detection probability						
Ψ (.), *p* (double/triple)	4	142.59	0.00	0.67	0.67	−67.09
Ψ (.), *p* (.)	3	144.05	1.46	0.33	1.00	−68.90
Occupancy						
Ψ (one), *p* (double/triple)	5	143.47	0.00	0.47	0.47	−66.42
Ψ (one, road), *p* (double/triple)	6	143.55	0.07	0.45	0.93	−65.33
Ψ (.), *p* (double/triple)	4	147.18	3.71	0.07	1.00	−69.38

*Note:* The relative best‐fit model structure of detection probability (*p*) was kept constant while modeling occupancy probability (Ψ). Double/triple = area under irrigated agriculture, one = area under open natural ecosystems, road = length of major road network.

**TABLE 4:** Additive models run to predict detection probability (*p*) and occupancy probability (Ψ) for sloth bear.

	*K* (#Parameters)	QAICc	ΔQAICc	QAICc weight	Cumulative weight	Quasi‐log likelihood
Detection probability						
Ψ (.), *p* (one)	4	82.06	0.00	0.37	0.37	−36.86
Ψ (.), *p* (one, kharif)	5	82.76	0.70	0.63	0.63	−36.12
Ψ (.), *p* (.)	3	82.93	0.87	0.87	0.87	−38.36
Ψ (.), *p* (kharif)	4	84.20	2.13	1.00	1.00	−37.92
Occupancy						
Ψ (.), *p* (one)	4	71.53	0.00	0.28	0.28	−31.59
Ψ (one, road), *p* (one)	6	71.61	0.07	0.27	0.55	−29.43
Ψ (road), *p* (one)	5	71.68	0.15	0.26	0.81	−30.57
Ψ (one), *p* (one)	5	72.26	0.73	0.19	1.00	−30.87

*Note:* Detection probability (*p*) was modeled by keeping the Null structure for occupancy probability (Ψ) constant. The relative best‐fit model structure of detection probability (*p*) was kept constant while modeling Occupancy probability (Ψ). Double/triple = area under irrigated agriculture, kharif = area under monsoonal agriculture, one = area under open natural ecosystems, road = length of major road network.

**TABLE 5:** Additive models run to predict detection probability (*p*) and occupancy probability (Ψ) for blackbuck.

	*K* (#Parameters)	QAICc	ΔQAICc	QAICc weight	Cumulative weight	Quasi‐log likelihood
Detection probability						
Ψ (.), *p* (exp, one, kharif)	6	228.71	0.00	0.53	0.53	−107.98
Ψ (.), *p* (one, kharif)	5	229.03	0.32	0.45	0.98	−109.25
Ψ (.), *p* (one)	4	237.38	8.67	0.01	0.99	−114.51
Ψ (.), *p* (exp, one)	5	237.44	8.73	0.00	0.99	−113.46
Ψ (.), *p* (exp, kharif)	5	239.13	10.42	0.00	1.00	−114.30
Ψ (.), *p* (kharif)	4	239.20	10.49	0.00	1.00	−115.42
Ψ (.), *p* (.)	3	247.01	18.30	0.00	1.00	−120.40
Ψ (.), *p* (exp)	4	247.36	18.65	0.00	1.00	−119.50
Occupancy						
Ψ (road), *p* (exp, one, kharif)	7	200.92	0.00	0.58	0.58	−92.95
Ψ (road, kharif), *p* (exp, one, kharif)	8	201.99	1.07	0.34	0.91	−92.34
Ψ (.), *p* (exp, one, kharif)	6	205.31	4.39	0.06	0.98	−96.28
Ψ (kharif), *p* (exp, one, kharif)	7	207.47	6.55	0.02	1.00	−96.23

*Note:* Detection probability (*p*) was modeled by keeping the Null structure for occupancy probability (Ψ) constant. The relative best‐fit model structure of detection probability (*p*) was kept constant while modeling occupancy probability (Ψ). Double/triple = area under irrigated agriculture, exp. = years of shepherding experience of the respondent, kharif = area under monsoonal agriculture, one = area under open natural ecosystems, road = length of major road network.

**FIGURE 5:**
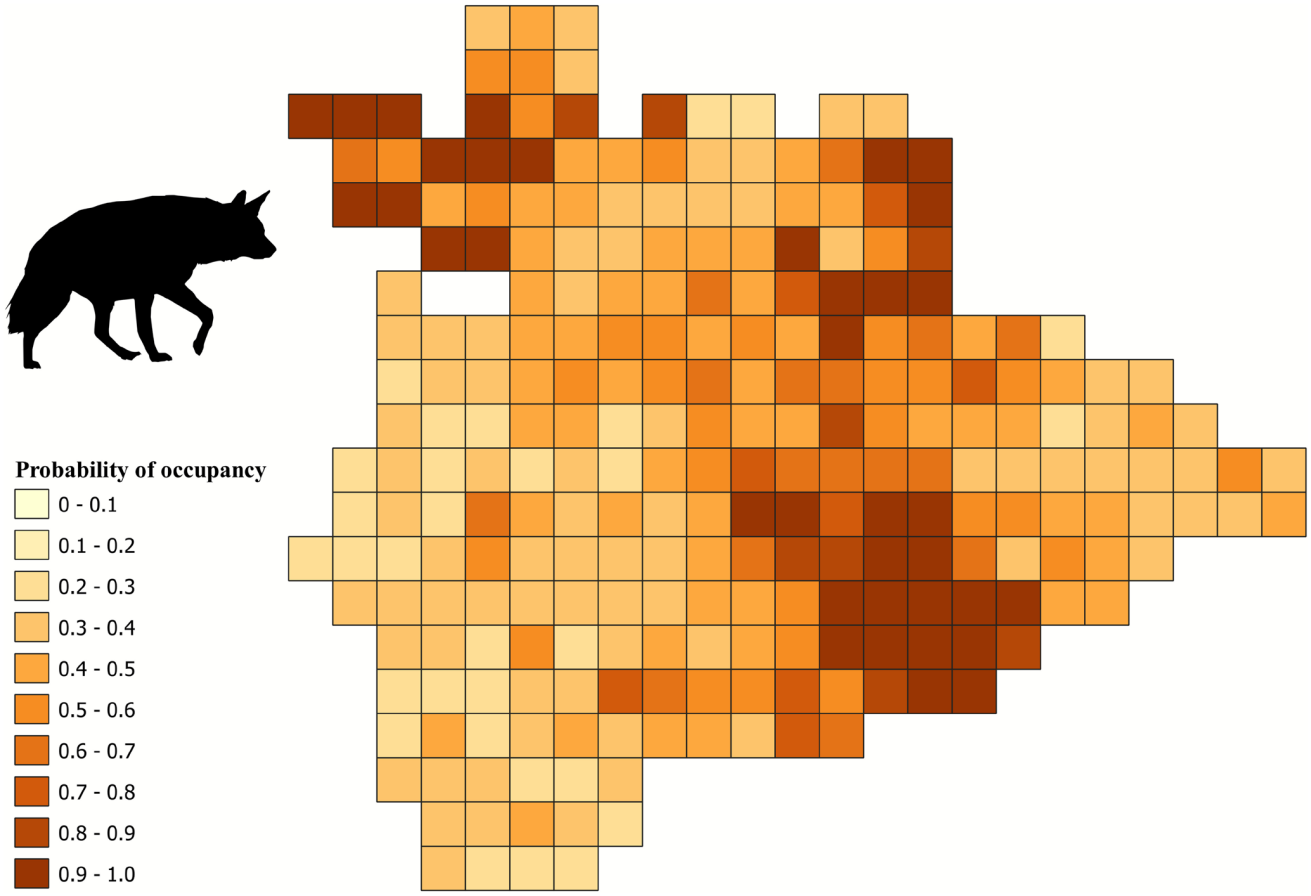
Map depicting the spatial distribution of occupancy probabilities of hyena in the study area.

**FIGURE 6:**
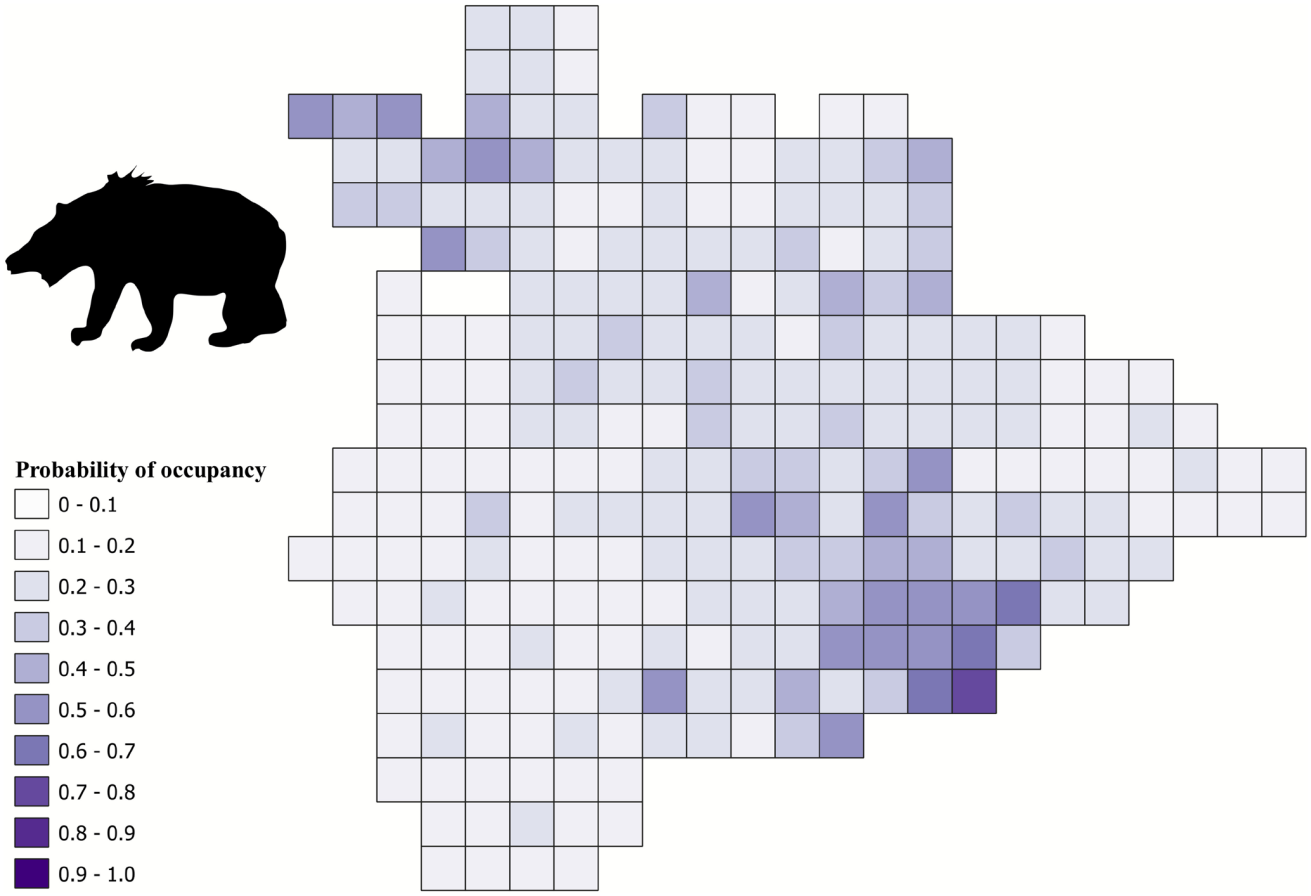
Map depicting the spatial distribution of occupancy probabilities of sloth bear in the study area.

**FIGURE 7:**
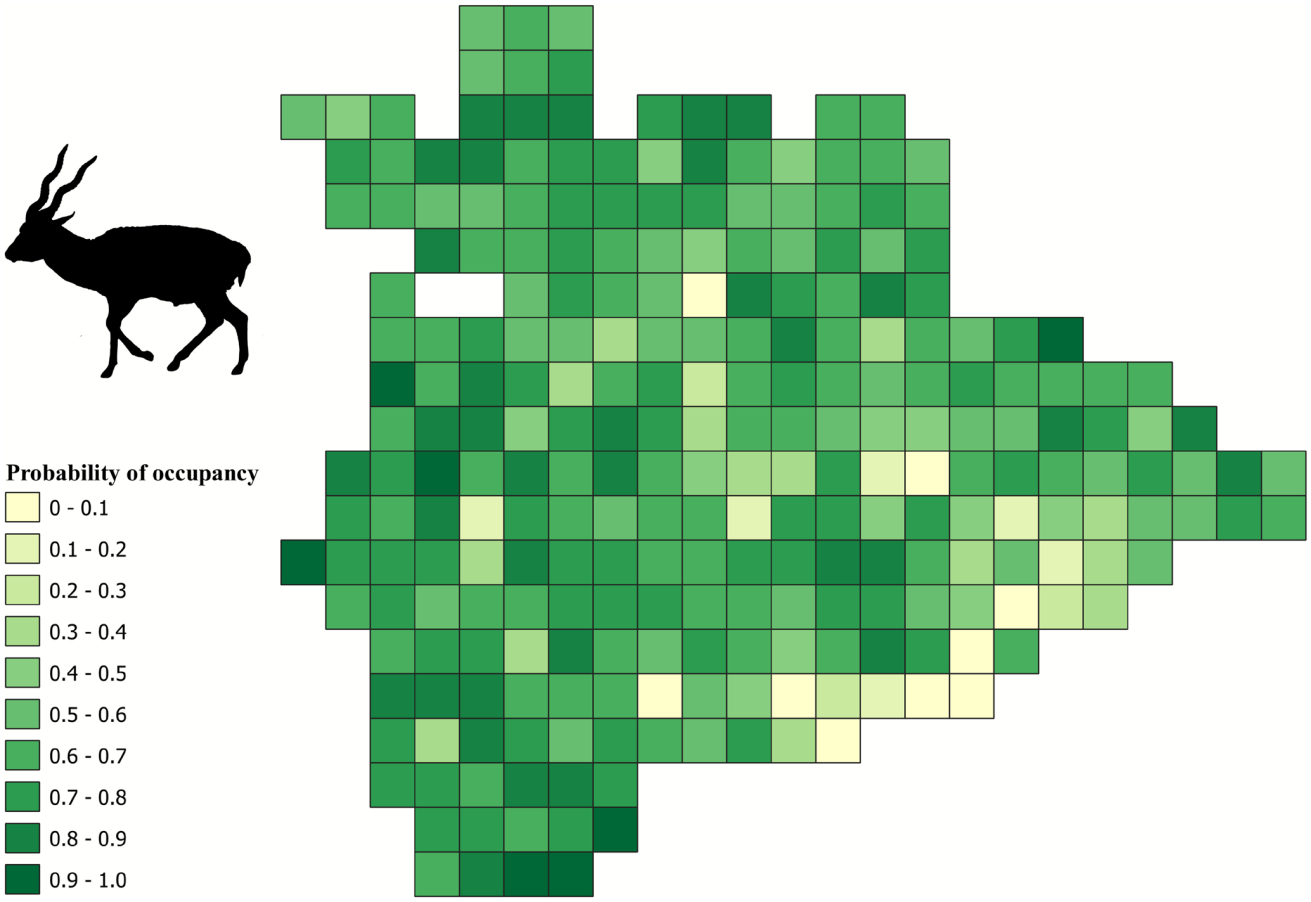
Map depicting the spatial distribution of occupancy probabilities of blackbuck in the study area.

Model‐averaged Ψ for hyena across the study area was 0.52 (SE 0.01, Figure 5). Model‐averaged Ψ for sloth bear across the study area was 0.26 (SE 0.01, Figure 6). Model‐averaged Ψ for blackbuck across the study area was 0.63 (SE 0.01, Figure 7). Area under irrigated agriculture best explained the detection probability of hyena (Table 3). Area of the grid under ONE best explained the detection probability of sloth bear (Table 4). Detection probability for blackbuck was best explained by the additive effect of respondents' experience, area of the grid under ONE, and area of the grid under monsoonal (*kharif*) cropping (Table 5).

Hyena presence was positively influenced by the extent of area under ONE (1.66, SE 1.58) and road length (1.05, SE 0.72) (Table 2). Livestock biomass and area under irrigated (*double/triple*) agriculture did not appear as good predictors for hyena occupancy (Table 2). Sloth bear occupancy was favored by the extent of area under ONE (1.06, SE 0.87) and the total road length (0.85, SE 0.41), but area under monsoonal (*kharif*) cropping was not a good predictor (Table 2). Blackbuck presence was positively influenced by the extent of area under the monsoonal (*kharif*) crop (1.30, SE 0.99) and negatively influenced by the total road length (−1.20, SE 0.40), while area under ONE appeared not to be a good predictor for blackbuck occupancy (Table 2).

## 4 Discussion

Wide‐ranging species populations in the semi‐arid agro‐pastoral landscapes of peninsular India are data deficient (Ghosh‐Harihar et al. 2019; Srivathsa et al. 2022), which weakens evidence‐based conservation governance in these human‐use regions (Sengupta et al. 2024). Our work produces baseline knowledge on the distribution of three large bodied species: striped hyena, sloth bear and blackbuck—known to depend on open ecosystems—from a representative landscape of the Deccan Peninsula. Our results also indicate the potential of agro‐pastoral lands in supporting populations of another globally “vulnerable” species, the Indian gray wolf (Hennelly et al. 2025) which is adapted to semi‐arid ONEs. This addresses a key gap in our understanding of which biophysical factors support ONE‐dependent wide‐ranging species in semi‐arid rural landscapes, while highlighting the potential of these neglected regions in conserving species populations.

### 4.1 Wildlife in Agro‐Pastoral “Wastelands”

We estimated occupancy for three large mammals—striped hyena, sloth bear and blackbuck—in north Karnataka's Koppal district. While our study design involved making a distinction between “certain” and “uncertain” detections for use of false positive models (Madsen et al. 2020; Miller et al. 2013; Pillay et al. 2014), we chose to use standard single‐season single‐species models (MacKenzie et al. 2018, 2002) while being conservative in constructing detection histories (refer to results section) because of non‐convergence issues.

Our findings show that blackbucks are more widespread (distributed across 63% of the region) compared to sloth bears and hyenas, which were distributed over 26% and 52% of the study area, respectively. Areas with high occupancy probabilities for blackbuck were scattered throughout Koppal district indicating that their spatial overlap with people was relatively higher (Figure 7). On the other hand, high‐occupancy cells for sloth bears and hyenas were concentrated in and around ONEs in the landscape indicating a lower spatial overlap with people (Figures 5 and 6). Blackbuck occurrence was favored by low‐intensity agriculture (monsoon cropping, “*kharif*”) and areas with low road connectivity (Table 2), a result aligned with findings from other human‐use landscapes (Asif and Modse 2016; Delu et al. 2024; Krishna et al. 2016). However, it did not show any relationship with the area under ONEs which are primarily in the form of rocky inselbergs with scrub vegetation. The presence of both sloth bears and hyenas, however, was positively influenced by ONEs (Table 2), which offer rugged hilly terrain for both species in a multi‐use landscape (Jangid et al. 2023; Puri et al. 2015; Singh et al. 2014).

Contrary to our predictions, irrigated cropping and monsoonal cropping were found to be poor predictors of hyena and sloth bear occupancy, respectively. However, both species show a strong preference for ONEs compared to other land uses (Puri et al. 2015; Singh et al. 2010). Consistent with previous studies, livestock biomass was not a good predictor for hyena presence, suggesting that carrion availability might not be driven by livestock biomass (Singh et al. 2010). Contrary to our a priori expectations that roads will negatively impact occupancy because of intensified human use, longer major road networks appeared to favor the occupancy of sloth bears and hyenas (Tables 1 and 2). This pattern may have been observed because major road networks in Koppal are denser in and around ONEs (coinciding with the presence of major towns) compared to the black cotton soil plains, owing to the soil morphology of the latter. While hyenas have been known to persist close to human habitation because of carrion availability (Panda et al. 2022; Singh et al. 2014) and sloth bear activity also has been seen to occur close to human habitation (Akhtar et al. 2004; Palei et al. 2020; Rot et al. 2023), we do not have enough causal evidence to conclude that major road networks support the occupancy of these species. Moreover, road characteristics (surroundings, traffic intensity, etc.) are also likely to influence how species use them (St‐Pierre et al. 2022) and further inquiry is needed to understand whether road networks in the study area facilitate species presence through, say, nocturnal movement (Dickie et al. 2022). Due to sparse data and high detection rates, we were unable to model occupancy for leopards and wolves, respectively. However, in the case of the latter, despite being conservative and treating uncertain detections as non‐detections, it was evident that wolves are widespread in the Koppal district and are present in both low‐intensity agricultural areas (*kharif*) as well as ONEs, a finding common to their populations in other regions of India (Habib et al. 2021; Khan et al. 2022; Majgaonkar et al. 2019; Mukherjee et al. 2021).

### 4.2 Agro‐Pastoral Lands as Shared Landscapes

It is noteworthy that not only is Koppal district devoid of Protected Areas (wildlife sanctuaries or national parks), but it also has a minor 7.64% of its area under the jurisdiction of the forest bureaucracy (https://aranya.gov.in/). However, at least four large mammal species (blackbuck, striped hyena, sloth bear, and wolves) are present in the matrix of private land tenure—in the form of agricultural lands—interspersed with land under the forest department jurisdiction, primarily in the form of different types of ONEs like rocky inselbergs, scrublands, and open savannas (Koulgi and Madhusudan 2024). ONEs in the form of private and public lands in this region are seasonally used for pastoralism and hence support human and livestock activity throughout the day. We argue that in addition to being adapted to semi‐arid rocky habitats, a combination of intermittent, seasonal anthropogenic use of ONEs, their rugged topography, and relative “intactness” over the years offers refugia for at least three of our focal species (striped hyena, sloth bear, and wolf) and allows opportunity for spatial segregation from human activity during the day (Grilo et al. 2019; Oeser et al. 2023; Schuette et al. 2013). Moreover, most large carnivores in human‐use landscapes modify activity patterns (Frey et al. 2020; Shamoon et al. 2018). Thus, the presence of seasonally used open agricultural lands around ONEs, which are devoid of human activity after daylight, may allow these species to persist outside Protected Areas in the absence of large wild prey (for striped hyena and wolf) and high‐quality food sources (for sloth bear) in ONEs (Johnson et al. 2020). For species like blackbuck, whose distribution encompasses large swathes of private land tenures, we argue that a mix of low‐intensity monsoonal agriculture and year‐round fallows, combined with low road connectivity, has enabled their populations to persist. Some of these fallow agricultural lands are also interspersed with alluvial streams, regionally called “*hallas*” (part of the dendritic water drainage pattern in this region), which dry post‐monsoon and likely offer refuge to blackbuck herds in open agricultural lands despite intermittent human activity (*IM pers. obs*.). Koppal district's relatively low rate of urbanization, as evidenced by a mere 1.35% increase in urban cover between 1991 and 2011 (Eswar and Roy 2018), may also have contributed to the persistence of these species. Since permanent pastures account for a scant proportion—3.23% of the district's geographical area—and are highly fragmented (Directorate of Economics and Statistics 2021), it is unlikely that they contribute to maintaining habitats for wide‐ranging species that were the focus of this study. While our findings show that the existing ONE‐agriculture matrix landscape supports large mammals, the structure and quality of these matrices are likely to determine source‐sink population dynamics (Gehr et al. 2017; Lamb et al. 2020; Nakamura et al. 2021) and fitness of individuals (Johnson et al. 2020; López‐Bao et al. 2019). Hence, our findings should not be construed as supporting ONE fragmentation merely because the species persist in ONE‐agricultural matrices, which is likely facilitated by altered activity patterns (Frey et al. 2020; Shamoon et al. 2018). Moreover, our results should also not be interpreted as a cost‐free coexistence between people and large‐bodied wildlife. Coexistence is a complex, multidimensional process (Nyhus 2016; Pooley et al. 2021), and the mere occurrence of a species cannot be interpreted as evidence of harmonious coexistence. Needs of pastoralism can negatively impact wildlife populations, say by pushing them out of high‐quality habitats (Okello 2005; Soofi et al. 2018). Even in Koppal, official records document 149 cases of crop loss, livestock loss, and human injury between 2007 and 2017 (data from Koppal Forest Division), and these figures are likely to be underreported. However, it is also noteworthy that the species occurrence data used in this study were entirely derived from the experiential knowledge of pastoralists and only a sustained co‐occurrence between them and large mammals could have allowed for that knowledge base to exist. Differential degrees of coexistence between pastoralists and wildlife have been similarly observed in other regions of the globe (Connolly et al. 2021; Kiffner et al. 2020; Mbise et al. 2020; Ogutu et al. 2017).

### 4.3 Pastoralism Central to Conservation of ONE Biodiversity

Most ONEs in India are in the form of working landscapes with approximately only 5% being inside protected areas (Madhusudan and Vanak 2023). Pastoralists are directly dependent on these ONEs to maintain seasonal mobility, a livelihood requirement, which then leads to them sharing these geographies with threatened wildlife (Kiffner et al. 2020; Madsen et al. 2020). The conservation of many species is hence highly dependent on coexistence with pastoralists. However, conversion of ONEs for “productive” uses, such as agriculture, green energy initiatives and afforestation, is increasingly threatening the viability of these lands to function as shared landscapes for livelihoods and wildlife conservation (Briske et al. 2025; Lahiri et al. 2023; Sheth et al. 2025). Continued access to landscape heterogeneity is a cornerstone for the persistence of semi‐arid systems (Hobbs et al. 2008), and the smaller the fragments of ONEs available for pastoralists, the higher the recursive use of these ecosystems for pastoralism. This can lead to pastoralist‐wildlife conflict intensification as has been observed in other semi‐arid regions (Hobbs et al. 2008). Moreover, with the loss of land sharing practices, the traditional ecological knowledge acquired and held by pastoralists is likely to diminish, even before it can be documented and applied to socially just conservation interventions in these landscapes. Our findings highlight how experiential knowledge of communities such as pastoralists can be applied in biodiversity conservation research to inform species ecology assessments in multi‐use landscapes (Madsen et al. 2020; Service et al. 2014).

### 4.4 Challenging the “Wasteland” Discourse

Much has been discussed about India's “wasteland” classification systems, especially the use of this terminology as a social and ecological category (Baka 2017; Watve et al. 2021), as well as how the discourse justifies diversion of lands for developmental purposes (Madhusudan and Vanak 2023). According to India's most updated wasteland atlas, Koppal district consists of three types of “wastelands”: land with dense/open scrub, underutilized degraded scrub, and barren rocky/stony waste, and these together cover 1151.92 km^2^, that is, 20% of the district (Department of Land Resources, National Remote Sensing Centre 2019). Our findings provide strong evidence against the use of these terminologies by demonstrating that not only do these “wastelands” or “marginal lands” function as habitat for endangered species in human‐dominated agrarian landscapes, but they are also not “underutilized” or “degraded” from the perspective of biodiversity conservation. These lands likely allow for a spatiotemporal separation between people and large‐bodied wildlife, thereby facilitating their co‐occurrence. Our work suggests that large mammal conservation in the Deccan Peninsula can be achieved within a land‐sharing framework if these mislabeled “wastelands” are recognized as ecologically important refugia and are appropriately conserved within a low‐intensity agricultural matrix.

## 5 Conclusion

Our findings add to the growing body of research highlighting the ecological role that ONEs play in the conservation of biodiversity (Misher and Vanak 2021; Srivathsa et al. 2020). Despite its ecological and geological significance, the Deccan peninsula of India has failed to attract as much conservation attention as the ONEs in western India (Athreya et al. 2013; Jayadevan et al. 2018; Misher and Vanak 2021), the savannas in western ghats and central India (Jathanna et al. 2015; Puri et al. 2023, 2015; Ramakrishnan et al. 1999; Sankaran 2009; Sankaran and McNaughton 1999) or high altitude grasslands in northern India (Bhatia et al. 2017; Kohli et al. 2021; Naidu et al. 2022; Namgail et al. 2007), possibly because of relatively lower species diversity (Srivathsa et al. 2023; Sudhakar Reddy et al. 2016) and higher fragmentation (Madhusudan and Vanak 2023). While we recognize that conservation monitoring of generalist species in native habitat‐production matrices with high human densities is presumably less exciting or lacks novelty, it remains undeniably important as human use regions currently constitute a large proportion of the earth's ice‐free surface (Ellis and Ramankutty 2008). If biodiversity assessments remain biased towards species‐rich and ostensibly “pristine” regions, this is likely to skew conservation efforts and render them inadequate to propose wildlife management strategies in multi‐use landscapes (Ghosal et al. 2013; Joppa and Pfaff 2009). Additionally, pastoral practices are increasingly being threatened by global climate change and local policy interventions (Sheth et al. 2025). Combined effect of such systemic biases against ONEs and people dependent on them could lead to the weakening of the socio‐ecological integrity of these human‐nature coupled systems and pave the way for unsustainable development planning in “mislabeled” and “misunderstood” landscapes, such as the Deccan Peninsula.

## Author Contributions

**Iravatee Majgaonkar:** conceptualization (lead), data curation (supporting), formal analysis (supporting), funding acquisition (lead), investigation (equal), methodology (lead), project administration (lead), resources (lead), validation (equal), writing – original draft (equal), writing – review and editing (equal). **Anish Paul:** data curation (supporting), formal analysis (lead), funding acquisition (supporting), investigation (equal), methodology (supporting), software (lead), validation (equal), visualization (lead), writing – original draft (equal), writing – review and editing (equal). **Sushma Sharma:** data curation (lead), investigation (supporting), project administration (supporting). **Indrajeet Ghorpade:** investigation (supporting), project administration (supporting), resources (supporting).

## Ethics Statement

Approval was obtained from the Human Subjects Committee of Centre for Wildlife Studies—Wildlife Conservation Society (India) (Application no. 2017‐001).

## Conflicts of Interest

The authors declare no conflicts of interest.

## Supporting information


**Figure S1:** Flowchart describing the methods used to consider species detection/non‐detection as “certain” and “uncertain” following an interview with a pastoralist. Certain detections were ascribed the value “2” and uncertain detections were ascribed the value “1.” All the non‐detections were denoted by “0.”

## Data Availability

All data supporting the findings and conclusion of this article has been made publicly available in the Zenodo data repository (https://doi.org/10.5281/zenodo.16955249). Any additional information, if required, will be made available by the corresponding author upon request.

## References

[ece372937-bib-0001] Akhtar, N. , H. S. Bargali , and N. P. S. Chauhan . 2004. “Sloth Bear Habitat Use in Disturbed and Unprotected Areas of Madhya Pradesh, India.” Ursu 15: 203–211. 10.2192/1537-6176(2004)015<0203:SBHUID>2.0.CO;2.

[ece372937-bib-0002] Asif, M. , and S. R. Modse . 2016. “The Distribution Pattern and Population of Blackbuck *Antilope cervicapra* Linnaeus in Bidar, Karnataka.” Indian Forester: 965–970. 10.36808/if/2016/v142i10/88157.

[ece372937-bib-0003] Athreya, V. , M. Odden , J. D. C. Linnell , J. Krishnaswamy , and U. Karanth . 2013. “Big Cats in Our Backyards: Persistence of Large Carnivores in a Human Dominated Landscape in India.” PLoS One 8: e57872. 10.1371/journal.pone.0057872. 23483933 PMC3590292

[ece372937-bib-0201] Athreya, V. , A. Srivathsa , M. Puri , K. K. Karanth , N. S. Kumar , and K. U. Karanth . 2015. “Spotted in the News: Using Media Reports to Examine Leopard Distribution, Depredation, and Management Practices outside Protected Areas in Southern India.” PLoS One 10: e0142647. 10.1371/journal.pone.0142647. 26556229 PMC4640542

[ece372937-bib-0004] Baka, J. 2017. “Making Space for Energy: Wasteland Development, Enclosures, and Energy Dispossessions.” Antipode 49: 977–996. 10.1111/anti.12219.

[ece372937-bib-0005] Baldi, G. , M. Texeira , O. A. Martin , H. R. Grau , and E. G. Jobbágy . 2017. “Opportunities Drive the Global Distribution of Protected Areas.” PeerJ 5: e2989. 10.7717/peerj.2989. 28229022 PMC5314958

[ece372937-bib-0006] Banerjee, D. , K. Chandra , C. Raghunathan , N. Singh , and D. Gupta . 2022. Faunal Diversity of Biogeographic Zones of India: Deccan Peninsula. Director, Zoological Surveyy of India.

[ece372937-bib-0204] Bargali, H. S. , N. Akhtar , and N. P. S. Chauhan . 2004. “Feeding Ecology of Sloth Bears in a Disturbed Area in Central India.” Ursus 15: 212–217. https://www.jstor.org/stable/3872974.

[ece372937-bib-0007] Barr, L. M. , R. L. Pressey , R. A. Fuller , D. B. Segan , E. McDonald‐Madden , and H. P. Possingham . 2011. “A New Way to Measure the World's Protected Area Coverage.” PLoS One 6: e24707. 10.1371/journal.pone.0024707. 21957458 PMC3177831

[ece372937-bib-0008] Bartoń, K. A. , T. Zwijacz‐Kozica , F. Zięba , A. Sergiel , and N. Selva . 2019. “Bears Without Borders: Long‐Distance Movement in Human‐Dominated Landscapes.” Global Ecology and Conservation 17: e00541. 10.1016/j.gecco.2019.e00541.

[ece372937-bib-0009] Bateman, P. W. , and P. A. Fleming . 2012. “Big City Life: Carnivores in Urban Environments.” Journal of Zoology 287: 1–23. 10.1111/j.1469-7998.2011.00887.x.

[ece372937-bib-0010] Bhatia, S. , S. M. Redpath , K. Suryawanshi , and C. Mishra . 2017. “The Relationship Between Religion and Attitudes Toward Large Carnivores in Northern India?” Human Dimensions of Wildlife 22: 30–42. 10.1080/10871209.2016.1220034.

[ece372937-bib-0011] Brashares, J. S. , P. Arcese , and M. K. Sam . 2001. “Human Demography and Reserve Size Predict Wildlife Extinction in West Africa. Proceedings of the Royal Society of London.” Series B: Biological Sciences 268: 2473–2478. 10.1098/rspb.2001.1815. PMC1088902 11747566

[ece372937-bib-0012] Briske, D. D. , P. G. M. Cromsigt , J. Davies , et al. 2025. “Pastoralism Can Mitigate Biodiversity Loss on Global Rangelands.” Bioscience: biaf158. 10.1093/biosci/biaf158. PMC12771521 41503404

[ece372937-bib-0013] Brittain, S. , M. N. Bata , P. D. Ornellas , E. J. Milner‐Gulland , and M. Rowcliffe . 2020. “Combining Local Knowledge and Occupancy Analysis for a Rapid Assessment of the Forest Elephant *Loxodonta cyclotis* in Cameroon's Timber Production Forests.” Oryx 54: 90–100. 10.1017/S0030605317001569.

[ece372937-bib-0014] Brown, M. B. , J. T. Fennessy , R. D. Crego , et al. 2023. “Ranging Behaviours Across Ecological and Anthropogenic Disturbance Gradients: A Pan‐African Perspective of Giraffe (*Giraffa* Spp.) Space Use.” Proceedings of the Royal Society B: Biological Sciences 290: 20230912. 10.1098/rspb.2023.0912. PMC10291724 37357852

[ece372937-bib-0015] Burnham, K. P. , and D. R. Anderson . 2002. Model Selection and Multimodel Inference: A Practical Information‐Theoretic Approach. 2nd ed. Springer‐Verlag.

[ece372937-bib-0016] Carter, N. H. , and J. D. C. Linnell . 2016. “Co‐Adaptation Is Key to Coexisting With Large Carnivores.” Trends in Ecology & Evolution 31: 575–578. 10.1016/j.tree.2016.05.006. 27377600

[ece372937-bib-0017] Connolly, E. , J. Allan , P. Brehony , et al. 2021. “Coexistence in an African Pastoral Landscape: Evidence That Livestock and Wildlife Temporally Partition Water Resources.” African Journal of Ecology 59: 696–711. 10.1111/aje.12869.

[ece372937-bib-0018] Cunningham, C. , and K. F. Beazley . 2018. “Changes in Human Population Density and Protected Areas in Terrestrial Global Biodiversity Hotspots, 1995–2015.” Land 7: 136. 10.3390/land7040136.

[ece372937-bib-0019] Delu, V. , D. Singh , S. Dookia , Priya , A. Godara , and V. Karwasra . 2024. “An Insight Into Population Structure and Seasonal Herd Pattern of Blackbuck *Antilope cervicapra* (Linnaeus, 1758) (Mammalia: Artiodactyla: Bovidae) in Semi‐Arid Region of Western Haryana, India.” Tropical Ecology 65: 92–102. 10.1007/s42965-023-00312-x.

[ece372937-bib-0020] Department of Animal Husbandry and Veterinary Services . 2019. 20th Livestock Census ‐ 2019 Karnataka Report. Government of Karnataka.

[ece372937-bib-0021] Department of Land Resources, National Remote Sensing Centre . 2019. “Wasteland Atlas of India Change Analysis Based on Temporal Satellite Data of 2008–09 and 2015–16.”

[ece372937-bib-0022] Dickie, M. , R. Serrouya , T. Avgar , et al. 2022. “Resource Exploitation Efficiency Collapses the Home Range of an Apex Predator.” Ecology 103: e3642. 10.1002/ecy.3642. 35066867

[ece372937-bib-0023] Directorate of Census Operations . 2011. District Census Handbook Koppal: Village and Town Directory (No. Series 30 Part 12 A). Government of India.

[ece372937-bib-0024] Directorate of Economics and Statistics . 2021. Karnataka at a Glance 2020–21. Government of Karnataka.

[ece372937-bib-0025] Doherty, P. F. , G. C. White , and K. P. Burnham . 2012. “Comparison of Model Building and Selection Strategies.” Journal Für Ornithologie 152: 317–323. 10.1007/s10336-010-0598-5.

[ece372937-bib-0026] Ellis, E. C. , and N. Ramankutty . 2008. “Putting People in the Map: Anthropogenic Biomes of the World.” Frontiers in Ecology and the Environment 6: 439–447. 10.1890/070062.

[ece372937-bib-0027] Eswar, M. , and A. K. Roy . 2018. “Urbanisation in Karnataka: Trend and Spatial Pattern.” Journal of Regional Development and Planning 7: 61–69.

[ece372937-bib-0028] Farhadinia, M. S. , P. J. Johnson , D. W. Macdonald , and L. T. B. Hunter . 2018. “Anchoring and Adjusting Amidst Humans: Ranging Behavior of Persian Leopards Along the Iran‐Turkmenistan Borderland.” PLoS One 13: e0196602. 10.1371/journal.pone.0196602. 29719005 PMC5931651

[ece372937-bib-0029] Fiske, I. , and R. Chandler . 2011. “Unmarked: An R Package for Fitting Hierarchical Models of Wildlife Occurrence and Abundance.” Journal of Statistical Software 43: 1–23. 10.18637/jss.v043.i10.

[ece372937-bib-0030] Frey, S. , J. P. Volpe , N. A. Heim , J. Paczkowski , and J. T. Fisher . 2020. “Move to Nocturnality Not a Universal Trend in Carnivore Species on Disturbed Landscapes.” Oikos 129: 1128–1140. 10.1111/oik.07251.

[ece372937-bib-0031] Gehr, B. , E. J. Hofer , S. Muff , et al. 2017. “A Landscape of Coexistence for a Large Predator in a Human Dominated Landscape.” Oikos 126: 1389–1399. 10.1111/oik.04182.

[ece372937-bib-0032] Ghosal, S. , V. R. Athreya , J. D. C. Linnell , and P. O. Vedeld . 2013. “An Ontological Crisis? A Review of Large Felid Conservation in India.” Biodiversity and Conservation 22: 2665–2681. 10.1007/s10531-013-0549-6.

[ece372937-bib-0033] Ghosh‐Harihar, M. , R. An , R. Athreya , et al. 2019. “Protected Areas and Biodiversity Conservation in India.” Biological Conservation 237: 114–124. 10.1016/j.biocon.2019.06.024.

[ece372937-bib-0034] Grilo, C. , P. M. Lucas , A. Fernández‐Gil , et al. 2019. “Refuge as Major Habitat Driver for Wolf Presence in Human‐Modified Landscapes.” Animal Conservation 22: 59–71. 10.1111/acv.12435.

[ece372937-bib-0035] Gubbi, S. , K. Sharma , and V. Kumara . 2020. “Every Hill Has Its Leopard: Patterns of Space Use by Leopards (*Panthera pardus*) in a Mixed Use Landscape in India.” PeerJ 8: e10072. 10.7717/peerj.10072. 33083134 PMC7548080

[ece372937-bib-0036] Habib, B. 2007. Ecology of Indian Wolf, Canis lupus pallipes sykes, 1831, and modeling its potential habitat in the great Indian bustard sanctuary, Maharashtra, India. Aligarh Muslim University.

[ece372937-bib-0037] Habib, B. , P. Ghaskadbi , S. Khan , Z. Hussain , and P. Nigam . 2021. “Not a Cakewalk: Insights Into Movement of Large Carnivores in Human‐Dominated Landscapes in India.” Ecology and Evolution 11: 1653–1666. 10.1002/ece3.7156. 33613996 PMC7882923

[ece372937-bib-0038] Hanumantha Rao, C. H. 1994. “Report of Technical Committee on Drought Prone Areas Program and Desert Development Program.” Indian Journal of Agricultural Economics 49.

[ece372937-bib-0039] Hennelly, L. M. , S. Khan , S. Sadhukhan , et al. 2025. “*Canis lupus* ssp. Pallipes.” The IUCN Red List of Threatened Species 2025 e.T223987953A223987966. 10.2305/IUCN.UK.2025-2.RLTS.T223987953A223987966.en.

[ece372937-bib-0040] Hobbs, N. T. , K. A. Galvin , C. J. Stokes , et al. 2008. “Fragmentation of Rangelands: Implications for Humans, Animals, and Landscapes.” Global Environmental Change 18: 776–785. 10.1016/j.gloenvcha.2008.07.011.

[ece372937-bib-0041] Hussain, M. S. , A. Mm , Y. Hm , S. Md , B. Us , and A. Ad . 2019. “Estimation of Body Weight and Dressed Weight in Different Sheep Breeds of Karnataka.” International Journal of Veterinary Science and Animal Husbandry 4: 10–14.

[ece372937-bib-0042] Jangid, A. K. , R. K. Sharma , and K. Ramesh . 2023. “Roads to the Hills: Potential Space Use Patterns of Sloth Bears and Leopards in Semiarid Landscape of Western India.” Mammal Study 48: 117–129. 10.3106/ms2022-0046.

[ece372937-bib-0043] Jathanna, D. , K. U. Karanth , N. S. Kumar , K. K. Karanth , and V. R. Goswami . 2015. “Patterns and Determinants of Habitat Occupancy by the Asian Elephant in the Western Ghats of Karnataka, India.” PLoS One 10: e0133233. 10.1371/journal.pone.0133233. 26207378 PMC4514602

[ece372937-bib-0044] Jayadevan, A. , S. Mukherjee , and A. T. Vanak . 2018. “Bush Encroachment Influences Nocturnal Rodent Community and Behaviour in a Semi‐Arid Grassland in Gujarat, India.” Journal of Arid Environments 153: 32–38. 10.1016/j.jaridenv.2017.12.009.

[ece372937-bib-0045] Jhala, Y. , S. Saini , S. Kumar , and Q. Qureshi . 2022. “Distribution, Status, and Conservation of the Indian Peninsular Wolf.” Frontiers in Ecology and Evolution 10. 10.3389/fevo.2022.814966.

[ece372937-bib-0046] Jhala, Y. V. , and R. H. Giles . 1991. “The Status and Conservation of the Wolf in Gujarat and Rajasthan, India.” Conservation Biology 5: 476–483.

[ece372937-bib-0205] Jhala, Y. V. , and K. Isvaran . 2016. “Behavioural Ecology of a Grassland Antelope, the Blackbuck *Antilope cervicapra*: Linking Habitat, Ecology and Behaviour.” In The Ecology of Large Herbivores in South and Southeast Asia, 151–176. Springer. 10.1007/978-94-017-7570-0_6.

[ece372937-bib-0047] Johnson, H. E. , D. L. Lewis , and S. W. Breck . 2020. “Individual and Population Fitness Consequences Associated With Large Carnivore Use of Residential Development.” Ecosphere 11: e03098. 10.1002/ecs2.3098.

[ece372937-bib-0048] Joppa, L. N. , and A. Pfaff . 2009. “High and Far: Biases in the Location of Protected Areas.” PLoS One 4: e8273. 10.1371/journal.pone.0008273. 20011603 PMC2788247

[ece372937-bib-0049] Joshi, M. 1995. “Patterns of Soil Respiration in a Temperate Grassland of Kumaun Himalaya, India.” Journal of Tropical Forest Science 8: 185–195.

[ece372937-bib-0050] Kabir, M. , S. Hameed , H. Ali , et al. 2017. “Habitat Suitability and Movement Corridors of Grey Wolf ( *Canis lupus* ) in Northern Pakistan.” PLoS One 12: e0187027. 10.1371/journal.pone.0187027. 29121089 PMC5679527

[ece372937-bib-0051] Kachel, S. , K. Anderson , and Q. Shokirov . 2022. “Predicting Carnivore Habitat Use and Livestock Depredation Risk With False‐Positive Multi‐State Occupancy Models.” Biological Conservation 271: 109588. 10.1016/j.biocon.2022.109588.

[ece372937-bib-0052] Kannan, P. , S. Salaria , S. Khan , et al. 2022. “Assessing Carnivore Occurrence and Community Attitudes Towards Wildlife in a Multi‐Use Arid Landscape Corridor.” Frontiers in Conservation Science 2. 10.3389/fcosc.2021.787431.

[ece372937-bib-0053] Karimi, A. , H. Yazdandad , and A. E. Reside . 2023. “Spatial Conservation Prioritization for Locating Protected Area Gaps in Iran.” Biological Conservation 279: 109902. 10.1016/j.biocon.2023.109902.

[ece372937-bib-0054] Khan, S. , S. Shrotriya , S. Sadhukhan , S. Lyngdoh , S. P. Goyal , and B. Habib . 2022. “Comparative Ecological Perspectives of Two Ancient Lineages of Gray Wolves: Woolly Wolf ( *Canis lupus chanco* ) and Indian Wolf ( *Canis lupus pallipes* ).” Frontiers in Ecology and Evolution 10. 10.3389/fevo.2022.775612.

[ece372937-bib-0055] Khan, S. , V. Vijayaraghavan , G. Talukdar , R. S. Kumar , and B. Habib . 2025. “Not All Those Who Wander Are Lost: Insights Into Movement Pattern of the Great Indian Bustard in the Deccan Landscape of India.” Ecology and Evolution 15: e71742. 10.1002/ece3.71742. 40625326 PMC12230192

[ece372937-bib-0056] Kiffner, C. , J. Kioko , J. Baylis , et al. 2020. “Long‐Term Persistence of Wildlife Populations in a Pastoral Area.” Ecology and Evolution 10: 10000–10016. 10.1002/ece3.6658. 33005359 PMC7520174

[ece372937-bib-0057] Kohli, M. , T. N. Mijiddorj , K. R. Suryawanshi , C. Mishra , B. Boldgiv , and M. Sankaran . 2021. “Grazing and Climate Change Have Site‐Dependent Interactive Effects on Vegetation in Asian Montane Rangelands.” Journal of Applied Ecology 58: 539–549. 10.1111/1365-2664.13781.

[ece372937-bib-0058] Koulgi, P. , and M. D. Madhusudan . 2024. “Mapping Landcover in India's Open Natural Ecosystems (ONEs).”

[ece372937-bib-0059] Krishna, Y. C. , A. Kumar , and K. Isvaran . 2016. “Wild Ungulate Decision‐Making and the Role of Tiny Refuges in Human‐Dominated Landscapes.” PLoS One 11: e0151748. 10.1371/journal.pone.0151748. 26985668 PMC4795686

[ece372937-bib-0060] Krishnadas, M. , M. Agarwala , S. Sridhara , and E. Eastwood . 2018. “Parks Protect Forest Cover in a Tropical Biodiversity Hotspot, but High Human Population Densities Can Limit Success.” Biological Conservation 223: 147–155. 10.1016/j.biocon.2018.04.034.

[ece372937-bib-0061] Lahiri, S. , A. Roy , and F. Fleischman . 2023. “Grassland Conservation and Restoration in India: A Governance Crisis.” Restoration Ecology 31: e13858. 10.1111/rec.13858.

[ece372937-bib-0062] Lamb, C. T. , A. T. Ford , B. N. McLellan , et al. 2020. “The Ecology of Human–Carnivore Coexistence.” Proceedings of the National Academy of Sciences 117: 17876–17883. 10.1073/pnas.1922097117. PMC7395549 32632004

[ece372937-bib-0063] López‐Bao, J. V. , M. Aronsson , J. D. C. Linnell , J. Odden , J. Persson , and H. Andrén . 2019. “Eurasian Lynx Fitness Shows Little Variation Across Scandinavian Human‐Dominated Landscapes.” Scientific Reports 9: 8903. 10.1038/s41598-019-45569-2. 31222101 PMC6586631

[ece372937-bib-0064] MacKenzie, D. I. , J. D. Nichols , G. B. Lachman , S. Droege , J. Royle , and C. A. Langtimm . 2002. “Estimating Site Occupancy Rates When Detection Probabilities Are Less Than One.” Ecology 83: 2248–2255. 10.1890/0012-9658(2002)083[2248:ESORWD]2.0.CO;2.

[ece372937-bib-0065] MacKenzie, D. I. , J. D. Nichols , J. A. Royle , K. H. Pollock , J. E. Hines , and L. L. Bailey . 2018. Occupancy Estimation and Modeling: Inferring Patterns and Dynamics of Species Occurrence. 2nd ed. Academic Press.

[ece372937-bib-0066] Madhusudan, M. D. , N. Sharma , R. Raghunath , et al. 2015. “Distribution, Relative Abundance, and Conservation Status of Asian Elephants in Karnataka, Southern India.” Biological Conservation 187: 34–40. 10.1016/j.biocon.2015.04.003.

[ece372937-bib-0067] Madhusudan, M. D. , and A. T. Vanak . 2023. “Mapping the Distribution and Extent of India's Semi‐Arid Open Natural Ecosystems.” Journal of Biogeography 50: 1377–1387. 10.1111/jbi.14471.

[ece372937-bib-0068] Madsen, E. K. , N. B. Elliot , E. E. Mjingo , et al. 2020. “Evaluating the Use of Local Ecological Knowledge (LEK) in Determining Habitat Preference and Occurrence of Multiple Large Carnivores.” Ecological Indicators 118: 106737. 10.1016/j.ecolind.2020.106737.

[ece372937-bib-0069] Mahato, A. K. R. , Ramakrishna , and M. Raziuddin . 2010. Status, Ecology & Behaviour of Antilope cervicapra (Linnaeus, 1758) in Proposed Community Reserve for Blackbuck, Ganjam District, Orissa, India. Director, Zoological Survey of India.

[ece372937-bib-0070] Majgaonkar, I. , A. Paul , S. Sharma , and I. Ghorpade . 2025. “Mislabeled and Misunderstood: Large Mammal Distribution Underscores Ecological Significance of Agro‐Pastoral “Wastelands” in India's Deccan Peninsula.” 10.5281/zenodo.16955249. PMC12795617 41531906

[ece372937-bib-0071] Majgaonkar, I. , S. Vaidyanathan , A. Srivathsa , S. Shivakumar , S. Limaye , and V. Athreya . 2019. “Land‐Sharing Potential of Large Carnivores in Human‐Modified Landscapes of Western India.” Conservation Science and Practice 1: e34. 10.1111/csp2.34.

[ece372937-bib-0072] Manoj Kumar, B. R. , T. Ganesh , and K. S. Seshadri . 2025. “Blackbuck Conservation in Fragmented Landscapes: Evaluating Habitat Use in and Around a Conservation Reserve.” Journal for Nature Conservation 88: 127040. 10.1016/j.jnc.2025.127040.

[ece372937-bib-0073] Mbise, F. P. , C. R. Jackson , R. Lyamuya , R. Fyumagwa , P. S. Ranke , and E. Røskaft . 2020. “Do Carnivore Surveys Match Reports of Carnivore Presence by Pastoralists? A Case of the Eastern Serengeti Ecosystem.” Global Ecology and Conservation 24: e01324. 10.1016/j.gecco.2020.e01324.

[ece372937-bib-0074] Miller, D. A. W. , J. D. Nichols , J. A. Gude , et al. 2013. “Determining Occurrence Dynamics When False Positives Occur: Estimating the Range Dynamics of Wolves From Public Survey Data.” PLoS One 8: e65808. 10.1371/journal.pone.0065808. 23840372 PMC3686827

[ece372937-bib-0075] Misher, C. , and A. T. Vanak . 2021. “Occupancy and Diet of the Indian Desert Fox *Vulpes vulpes pusilla* in a *Prosopis juliflora* Invaded Semi‐Arid Grassland.” Wildlife Biology 2021: wlb.00781. 10.2981/wlb.00781.

[ece372937-bib-0076] Mohammadi, A. , K. Almasieh , D. Nayeri , et al. 2021. “Identifying Priority Core Habitats and Corridors for Effective Conservation of Brown Bears in Iran.” Scientific Reports 11: 1044. 10.1038/s41598-020-79970-z. 33441776 PMC7806652

[ece372937-bib-0077] Mohammadi, A. , C. Lunnon , R. J. Moll , et al. 2021. “Contrasting Responses of Large Carnivores to Land Use Management Across an Asian Montane Landscape in Iran.” Biodiversity and Conservation 30: 4023–4037. 10.1007/s10531-021-02290-9.

[ece372937-bib-0078] Mukherjee, T. , I. Chongder , S. Ghosh , et al. 2021. “Indian Grey Wolf and Striped Hyaena Sharing From the Same Bowl: High Niche Overlap Between Top Predators in a Human‐Dominated Landscape.” Global Ecology and Conservation 28: e01682. 10.1016/j.gecco.2021.e01682.

[ece372937-bib-0079] Mundinamani, V. I. , M. D. Gouri , K. Prasad , and V. M. Patil . 2024. “Physical and Morphometric Characteristics of Unidentified Cattle Breed of Northern Karnataka Region of India.” Indian Journal of Dairy Science 77: 354–361.

[ece372937-bib-0202] Naha, D. , S. Dash , C. Kupferman , J. Beasley , and S. Sathyakumar . 2021. “Movement Behavior of a Solitary Large Carnivore Within a Hotspot of Human‐Wildlife Conflicts in India.” Scientific Reports 11: 3862. 10.1038/s41598-021-83262-5. 33594130 PMC7887241

[ece372937-bib-0080] Naidu, D. G. T. , S. Roy , and S. Bagchi . 2022. “Loss of Grazing by Large Mammalian Herbivores Can Destabilize the Soil Carbon Pool.” Proceedings of the National Academy of Sciences 119: e2211317119. 10.1073/pnas.2211317119. PMC9618051 36252005

[ece372937-bib-0081] Nakamura, M. , H. Rio‐Maior , R. Godinho , F. Petrucci‐Fonseca , and F. Álvares . 2021. “Source‐Sink Dynamics Promote Wolf Persistence in Human‐Modified Landscapes: Insights From Long‐Term Monitoring.” Biological Conservation 256: 109075. 10.1016/j.biocon.2021.109075.

[ece372937-bib-0082] Namgail, T. , Y. V. Bhatnagar , C. Mishra , and S. Bagchi . 2007. “Pastoral Nomads of the Indian Changthang: Production System, Landuse and Socioeconomic Changes.” Human Ecology 35: 497–504. 10.1007/s10745-006-9107-0.

[ece372937-bib-0083] Nyhus, P. J. 2016. “Human–Wildlife Conflict and Coexistence.” Annual Review of Environment and Resources 41: 143–171. 10.1146/annurev-environ-110615-085634.

[ece372937-bib-0084] Odden, M. , V. Athreya , S. Rattan , and J. D. C. Linnell . 2014. “Adaptable Neighbours: Movement Patterns of GPS‐Collared Leopards in Human Dominated Landscapes in India.” PLoS One 9: e112044. 10.1371/journal.pone.0112044. 25390067 PMC4229117

[ece372937-bib-0085] Oeser, J. , M. Heurich , S. Kramer‐Schadt , et al. 2023. “Prerequisites for Coexistence: Human Pressure and Refuge Habitat Availability Shape Continental‐Scale Habitat Use Patterns of a Large Carnivore.” Landscape Ecology 38: 1713–1728. 10.1007/s10980-023-01645-7.

[ece372937-bib-0086] Ogutu, J. O. , B. Kuloba , H.‐P. Piepho , and E. Kanga . 2017. “Wildlife Population Dynamics in Human‐Dominated Landscapes Under Community‐Based Conservation: The Example of Nakuru Wildlife Conservancy, Kenya.” PLoS One 12: e0169730. 10.1371/journal.pone.0169730. 28103269 PMC5245813

[ece372937-bib-0087] Okello, M. M. 2005. “Land Use Changes and Human–Wildlife Conflicts in the Amboseli Area, Kenya.” Human Dimensions of Wildlife 10: 19–28. 10.1080/10871200590904851.

[ece372937-bib-0088] Packer, C. , A. Loveridge , S. Canney , et al. 2013. “Conserving Large Carnivores: Dollars and Fence.” Ecology Letters 16: 635–641. 10.1111/ele.12091. 23461543

[ece372937-bib-0089] Palei, H. S. , S. Debata , and H. K. Sahu . 2020. “Diet of Sloth Bear in an Agroforest Landscape in Eastern India.” Agroforestry Systems 94: 269–279. 10.1007/s10457-019-00389-1.

[ece372937-bib-0090] Palfrey, R. , J. A. Oldekop , and G. Holmes . 2022. “Privately Protected Areas Increase Global Protected Area Coverage and Connectivity.” Nature Ecology & Evolution 6: 730–737. 10.1038/s41559-022-01715-0. 35393602

[ece372937-bib-0091] Panda, D. , S. Mohanty , T. Suryan , P. Pandey , H. Lee , and R. Singh . 2022. “High Striped Hyena Density Suggests Coexistence With Humans in an Agricultural Landscape, Rajasthan.” PLoS One 17: e0266832. 10.1371/journal.pone.0266832. 35507591 PMC9067646

[ece372937-bib-0092] Paul, A. , N. Kumar , T. Mukherjee , A. K. Chhetri , and A. Kshettry . 2024. “Rosettes in a Matrix: Predicting Spatial Variation in Density of a Large Felid in a Forest‐Production Mosaic.” Integrative Conservation 3: 426–437. 10.1002/inc3.71.

[ece372937-bib-0093] Petracca, L. S. , J. L. Frair , J. B. Cohen , et al. 2018. “Robust Inference on Large‐Scale Species Habitat Use With Interview Data: The Status of Jaguars Outside Protected Areas in Central America.” Journal of Applied Ecology 55: 723–734. 10.1111/1365-2664.12972.

[ece372937-bib-0094] Pillay, R. , D. A. W. Miller , J. E. Hines , A. A. Joshi , and M. D. Madhusudan . 2014. “Accounting for False Positives Improves Estimates of Occupancy From Key Informant Interviews.” Diversity and Distributions 20: 223–235. 10.1111/ddi.12151.

[ece372937-bib-0095] Pooley, S. , S. Bhatia , and A. Vasava . 2021. “Rethinking the Study of Human–Wildlife Coexistence.” Conservation Biology 35: 784–793. 10.1111/cobi.13653. 33044026 PMC8246872

[ece372937-bib-0096] Puri, M. , A. J. Marx , H. P. Possingham , K. A. Wilson , K. K. Karanth , and B. A. Loiselle . 2022. “An Integrated Approach to Prioritize Restoration for Carnivore Conservation in Shared Landscapes.” Biological Conservation 273: 109697. 10.1016/j.biocon.2022.109697.

[ece372937-bib-0097] Puri, M. , A. Srivathsa , K. K. Karanth , N. S. Kumar , and K. U. Karanth . 2015. “Multiscale Distribution Models for Conserving Widespread Species: The Case of Sloth Bear *Melursus ursinus* in India.” Diversity and Distributions 21: 1087–1100. 10.1111/ddi.12335.

[ece372937-bib-0098] Puri, M. , A. Srivathsa , K. K. Karanth , I. Patel , and N. S. Kumar . 2023. “Safe Space in the Woods: Mechanistic Spatial Models for Predicting Risks of Human–Bear Conflicts in India.” Biotropica 55: 504–516. 10.1111/btp.13204.

[ece372937-bib-0099] R Core Team . 2024. “R: A Language and Environment for Statistical Computing.”

[ece372937-bib-0100] Rahmani, A. R. 1990. “Distribution, Density, Group Size and Conservation of the Indian Gazelle or Chinkara GazeHa Bennetti (Sykes 1831) in Rajasthan, India.” Biological Conservation 51: 177–189.

[ece372937-bib-0101] Rahmani, A. R. , and R. G. Soni . 1997. “Avifaunal Changes in the Indian Thar Desert.” Journal of Arid Environments 36: 687–703. 10.1006/jare.1996.0242.

[ece372937-bib-0102] Ramakrishnan, U. , R. G. Coss , and N. W. Pelkey . 1999. “Tiger Decline Caused by the Reduction of Large Ungulate Prey: Evidence From a Study of Leopard Diets in Southern India.” Biological Conservation 89: 113–120. 10.1016/S0006-3207(98)00159-1.

[ece372937-bib-0103] Ratnam, J. , W. J. Bond , R. J. Fensham , et al. 2011. “When Is a “Forest” a Savanna, and Why Does It Matter?” Global Ecology and Biogeography 20: 653–660. 10.1111/j.1466-8238.2010.00634.x.

[ece372937-bib-0104] Ratnam, J. , K. W. Tomlinson , D. N. Rasquinha , and M. Sankaran . 2016. “Savannahs of Asia: Antiquity, Biogeography, and an Uncertain Future.” Philosophical Transactions of the Royal Society of London. Series B, Biological Sciences 371: 20150305. 10.1098/rstb.2015.0305. 27502371 PMC4978864

[ece372937-bib-0105] Ratnayeke, S. , F. T. van Manen , and U. K. G. K. Padmalal . 2007. “Home Ranges and Habitat Use of Sloth Bears *Melursus ursinus inornatus* in Wasgomuwa National Park, Sri Lanka.” Wildlife Biology 13: 272–284. 10.2981/0909-6396(2007)13%5B272:HRAHUO%5D2.0.CO;2.

[ece372937-bib-0106] Ratnayeke, S. , F. T. van Manen , R. Pieris , and V. S. J. Pragash . 2007. “Landscape Characteristics of Sloth Bear Range in Sri Lanka.” Ursu 18: 189–202. 10.2192/1537-6176(2007)18%5B189:LCOSBR%5D2.0.CO;2.

[ece372937-bib-0107] Rezaei, S. , A. Mohammadi , S. Malakoutikhah , and R. Khosravi . 2022. “Combining Multiscale Niche Modeling, Landscape Connectivity, and Gap Analysis to Prioritize Habitats for Conservation of Striped Hyaena ( *Hyaena hyaena* ).” PLoS One 17: e0260807. 10.1371/journal.pone.0260807. 35143518 PMC8830629

[ece372937-bib-0108] Riedel, N. , D. Q. Fuller , N. Marwan , et al. 2021. “Monsoon Forced Evolution of Savanna and the Spread of Agro‐Pastoralism in Peninsular India.” Scientific Reports 11: 9032. 10.1038/s41598-021-88550-8. 33907218 PMC8079367

[ece372937-bib-0109] Ripple, W. J. , J. A. Estes , R. L. Beschta , et al. 2014. “Status and Ecological Effects of the World's Largest Carnivores.” Science 343: 1241484. 10.1126/science.1241484. 24408439

[ece372937-bib-0110] Ripple, W. J. , T. M. Newsome , C. Wolf , et al. 2015. “Collapse of the World's Largest Herbivores.” Science Advances 1: e1400103. 10.1126/sciadv.1400103. 26601172 PMC4640652

[ece372937-bib-0111] Rot, J. , A. K. Jangid , C. P. Singh , and N. A. Dharaiya . 2023. “Escaping Neobiota: Habitat Use and Avoidance by Sloth Bears in Jessore Sloth Bear Sanctuary India.” Trees, Forests and People 13: 100400. 10.1016/j.tfp.2023.100400.

[ece372937-bib-0112] Royle, J. A. , and W. A. Link . 2006. “Generalized Site Occupancy Models Allowing for False Positive and False Negative Errors.” Ecology 87: 835–841. 10.1890/0012-9658(2006)87%5B835:GSOMAF%5D2.0.CO;2. 16676527

[ece372937-bib-0113] Sadhukhan, S. , S. Khan , and B. Habib . 2024. “Silencing the Call of the Wild – Howling Behaviour and Responses of the Wolf to Anthropocene in India.” Animal Conservation 27: 98–111. 10.1111/acv.12881.

[ece372937-bib-0114] Sankaran, M. 2009. “Diversity Patterns in Savanna Grassland Communities: Implications for Conservation Strategies in a Biodiversity Hotspot.” Biodiversity and Conservation 18: 1099–1115. 10.1007/s10531-008-9519-9.

[ece372937-bib-0115] Sankaran, M. , and S. J. McNaughton . 1999. “Determinants of Biodiversity Regulate Compositional Stability of Communities.” Nature 401: 691–693. 10.1038/44368.

[ece372937-bib-0116] Sankaran, M. , and J. Ratnam . 2013. “African and Asian Savannas.” In Encyclopedia of Biodiversity, 58–74. Elsevier. 10.1016/B978-0-12-384719-5.00355-5.

[ece372937-bib-0117] Sayre, R. , D. Karagulle , C. Frye , et al. 2020. “An Assessment of the Representation of Ecosystems in Global Protected Areas Using New Maps of World Climate Regions and World Ecosystems.” Global Ecology and Conservation 21: e00860. 10.1016/j.gecco.2019.e00860.

[ece372937-bib-0118] Schuette, P. , S. Creel , and D. Christianson . 2013. “Coexistence of African Lions, Livestock, and People in a Landscape With Variable Human Land Use and Seasonal Movements.” Biological Conservation 157: 148–154. 10.1016/j.biocon.2012.09.011.

[ece372937-bib-0119] Sengupta, A. , M. Bhan , S. Bhatia , A. Joshi , S. Kuriakose , and K. S. Seshadri . 2024. “Realizing “30 × 30” in India: The Potential, the Challenges, and the Way Forward.” Conservation Letters 17: e13004. 10.1111/conl.13004.

[ece372937-bib-0120] Service, C. N. , M. S. Adams , K. A. Artelle , P. Paquet , L. V. Grant , and C. T. Darimont . 2014. “Indigenous Knowledge and Science Unite to Reveal Spatial and Temporal Dimensions of Distributional Shift in Wildlife of Conservation Concern.” PLoS One 9: e101595. 10.1371/journal.pone.0101595. 25054635 PMC4108310

[ece372937-bib-0121] Shamoon, H. , R. Maor , D. Saltz , and T. Dayan . 2018. “Increased Mammal Nocturnality in Agricultural Landscapes Results in Fragmentation due to Cascading Effects.” Biological Conservation 226: 32–41. 10.1016/j.biocon.2018.07.028.

[ece372937-bib-0122] Sheth, A. , M. Kohli , J. Ratnam , V. Saberwal , and M. Sankaran . 2025. “Towards Greener Pastures: An Overview of Pastoral Economics, Policies and Ecologies in India.” Economic and Political Weekly 60: 57–65.

[ece372937-bib-0123] Siddalingamurthy, H. K. , R. M. Sreesujatha , and S. S. Manjunatha . 2017. “Determination of Regression Model for Prediction of Body Weight in Local Goats of Mandya District in Karnataka.” IJIRES 4: 182–184.

[ece372937-bib-0124] Simlai, T. , and C. Sandbrook . 2025. “The Gendered Forest: Digital Surveillance Technologies for Conservation and Gender‐Environment Relationships.” Environment and Planning F 4: 157–174. 10.1177/26349825241283837.

[ece372937-bib-0125] Singh, G. , R. Babu , P. Narain , L. S. Bhushan , and I. P. Abrol . 1992. “Soil Erosion Rates in India.” Journal of Soil and Water Conservation 47: 97–99. 10.1080/00224561.1992.12456680.

[ece372937-bib-0203] Singh, M. , and H. N. Kumara . 2006. “Distribution, Status and Conservation of Indian Gray Wolf (*Canis lupus pallipes*) in Karnataka, India.” Journal of Zoology 270, no. 1: 164–169. 10.1111/j.1469-7998.2006.00103.x.

[ece372937-bib-0126] Singh, P. , A. Gopalaswamy , and K. Karanth . 2010. “Factors Influencing Densities of Striped Hyenas (*Hyaena hyaena*) in Arid Regions of India.” Journal of Mammalogy 91: 1152–1159. 10.2307/40925721.

[ece372937-bib-0127] Singh, R. , Q. Qureshi , K. Sankar , P. R. Krausman , S. P. Goyal , and K. L. Nicholson . 2014. “Population Density of Striped Hyenas in Relation to Habitat in a Semi‐Arid Landscape, Western India.” Acta Theriologica 59: 521–527. 10.1007/s13364-014-0187-8.

[ece372937-bib-0128] Soofi, M. , A. Ghoddousi , T. Zeppenfeld , et al. 2018. “Livestock Grazing in Protected Areas and Its Effects on Large Mammals in the Hyrcanian Forest, Iran.” Biological Conservation 217: 377–382. 10.1016/j.biocon.2017.11.020.

[ece372937-bib-0129] Srivathsa, A. , A. Banerjee , S. Banerjee , et al. 2022. “Chasms in Charismatic Species Research: Seventy Years of Carnivore Science and Its Implications for Conservation and Policy in India.” Biological Conservation 273: 109694. 10.1016/j.biocon.2022.109694.

[ece372937-bib-0130] Srivathsa, A. , I. Majgaonkar , S. Sharma , et al. 2020. “Opportunities for Prioritizing and Expanding Conservation Enterprise in India Using a Guild of Carnivores as Flagships.” Environmental Research Letters 15: 064009. 10.1088/1748-9326/ab7e50.

[ece372937-bib-0131] Srivathsa, A. , D. Vasudev , T. Nair , et al. 2023. “Prioritizing India's Landscapes for Biodiversity, Ecosystem Services and Human Well‐Being.” Nature Sustainability 6: 568–577. 10.1038/s41893-023-01063-2.

[ece372937-bib-0132] St‐Pierre, F. , P. Drapeau , and M.‐H. St‐Laurent . 2022. “Stairway to Heaven or Highway to Hell? How Characteristics of Forest Roads Shape Their Use by Large Mammals in the Boreal Forest.” Forest Ecology and Management 510: 120108. 10.1016/j.foreco.2022.120108.

[ece372937-bib-0133] Sudhakar Reddy, C. , C. S. Jha , V. K. Dadhwal , et al. 2016. “Quantification and Monitoring of Deforestation in India Over Eight Decades (1930–2013).” Biodiversity and Conservation 25: 93–116. 10.1007/s10531-015-1033-2.

[ece372937-bib-0134] Suraci, J. P. , B. A. Nickel , and C. C. Wilmers . 2020. “Fine‐Scale Movement Decisions by a Large Carnivore Inform Conservation Planning in Human‐Dominated Landscapes.” Landscape Ecology 35: 1635–1649. 10.1007/s10980-020-01052-2.

[ece372937-bib-0135] Tian, H. , K. Banger , T. Bo , and V. K. Dadhwal . 2014. “History of Land Use in India During 1880–2010: Large‐Scale Land Transformations Reconstructed From Satellite Data and Historical Archives.” Global and Planetary Change 121: 78–88. 10.1016/j.gloplacha.2014.07.005.

[ece372937-bib-0136] Valeix, M. , G. Hemson , A. J. Loveridge , G. Mills , and D. W. Macdonald . 2012. “Behavioural Adjustments of a Large Carnivore to Access Secondary Prey in a Human‐Dominated Landscape.” Journal of Applied Ecology 49: 73–81. 10.1111/j.1365-2664.2011.02099.x.

[ece372937-bib-0137] Warrier, R. , B. R. Noon , and L. Bailey . 2020. “Agricultural Lands Offer Seasonal Habitats to Tigers in a Human‐Dominated and Fragmented Landscape in India.” Ecosphere 11: e03080. 10.1002/ecs2.3080.

[ece372937-bib-0138] Watson, J. E. M. , K. R. Jones , R. A. Fuller , et al. 2016. “Persistent Disparities Between Recent Rates of Habitat Conversion and Protection and Implications for Future Global Conservation Targets.” Conservation Letters 9: 413–421. 10.1111/conl.12295.

[ece372937-bib-0139] Watve, A. , V. Athreya , and I. Majgaonkar . 2021. “The Need to Overhaul Wasteland Classification Systems in India | Economic and Political Weekly.” Economic and Political Weekly 56: 36–40.

[ece372937-bib-0140] Whitehead, J. 2010. “John Locke and the Governance of India's Landscape: The Category of Wasteland in Colonial Revenue and Forest Legislation.” Economic and Political Weekly 45: 83–93.

[ece372937-bib-0141] Woodroffe, R. 2000. “Predators and People: Using Human Densities to Interpret Declines of Large Carnivores.” Animal Conservation 3: 165–173. 10.1111/j.1469-1795.2000.tb00241.x.

