# Correction to “Mislabeled and Misunderstood: Large Mammal Distribution Underscores Ecological Significance of Agro‐Pastoral “Wastelands” in India's Deccan Peninsula”

**DOI:** 10.1002/ece3.73195

**Published:** 2026-02-26

Majgaonkar, I., A. Paul, S. Sharma, and I. Ghorpade. 2026. “Mislabeled and Misunderstood: Large Mammal Distribution Underscores Ecological Significance of Agro‐Pastoral “Wastelands” in India's Deccan Peninsula.” *Ecology and Evolution* 16, no. 1: e72937. https://doi.org/10.1002/ece3.72937.

In paragraph 2 of the section 2.4 “Covariate Extraction,” the line “For land cover classes, we used 2015–2016 1:50,000 LULC raster from Indian Space Research Organization's Bhuvan portal (https://bhuvan‐app1.nrsc.gov.in/thematic/thematic/index.php).” was incorrect. This should have read “For land cover classes, we used 2015–2016 1:250,000 LULC raster from Indian Space Research Organization's Bhuvan portal (https://bhuvan‐app1.nrsc.gov.in/thematic/thematic/index.php).”

The scale of the map used to extract the land cover data for the study site was mentioned incorrectly in the manuscript. While this does not create any difference in the subsequent text and analysis, we believe it would be fair to correct this error for the benefit of the readers and reproducibility of the results.

