# Supplementary material for: Mislabeled and Misunderstood: Large Mammal Distribution Underscores Ecological Significance of Agro‐Pastoral “Wastelands” in India's Deccan Peninsula
Source: Ecol Evol. 2026 Jan 12;16(1):e72937. doi: 10.1002/ece3.72937 (PMC12795617; doi:10.1002/ece3.72937)

Supplementary Figure 1: Flowchart describing the methods used to consider species detection/non-detection as ‘certain’ and ‘uncertain’ following an interview with a pastoralist. Certain detections were ascribed the value ‘2’ and uncertain detections were ascribed the value ‘1’. All the non-detections were denoted by ‘0’.

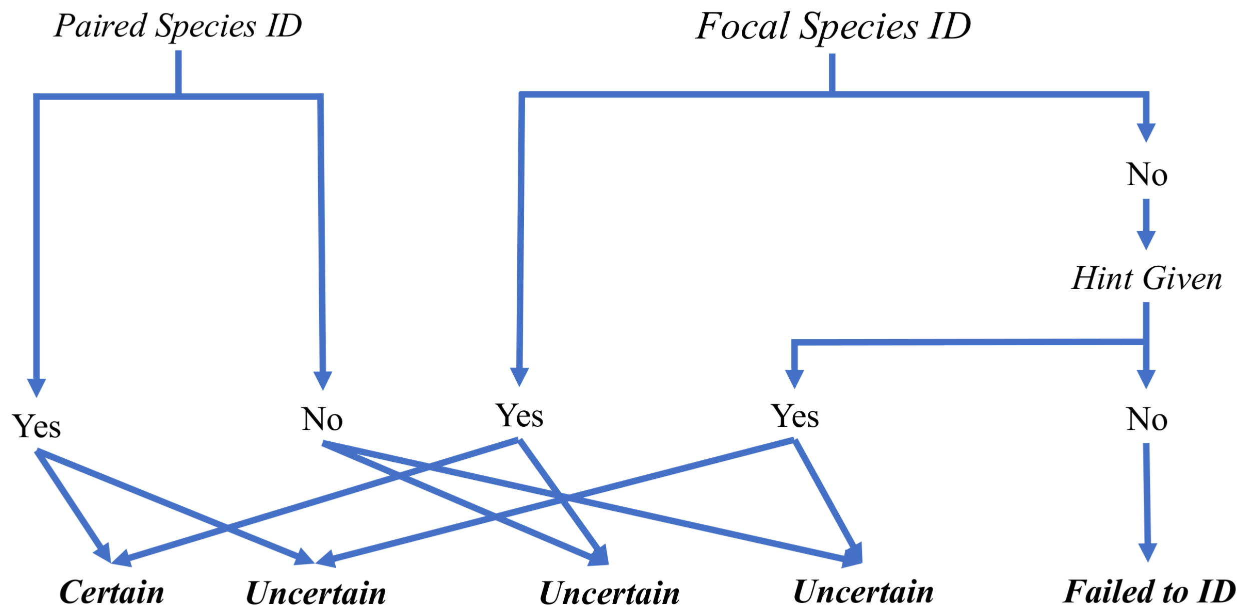

Supplement: Supplementary file 1 — Figure S1: Flowchart describing the methods used to consider species detection/non‐detection as “certain” and “uncertain” following an interview with a pastoralist. Certain detections were ascribed the value “2” and uncertain detections were ascribed the value “1.” All the non‐detections were denoted by “0.” [file ECE3-16-e72937-s001.pdf]
